# Characterization of *Staphylococcus* and *Corynebacterium* Clusters in the Human Axillary Region

**DOI:** 10.1371/journal.pone.0070538

**Published:** 2013-08-12

**Authors:** Chris Callewaert, Frederiek-Maarten Kerckhof, Michael S. Granitsiotis, Mireille Van Gele, Tom Van de Wiele, Nico Boon

**Affiliations:** 1 Laboratory of Microbial Ecology and Technology, Ghent University, Gent, Belgium; 2 Helmholtz Center Munich, German Research Center for Environmental Health (GmbH), Research Unit Environmental Genomics, Neuherberg, Germany; 3 Department of Dermatology, Ghent University Hospital, Gent, Belgium; University Medical Center Utrecht, Netherlands

## Abstract

The skin microbial community is regarded as essential for human health and well-being, but likewise plays an important role in the formation of body odor in, for instance, the axillae. Few molecular-based research was done on the axillary microbiome. This study typified the axillary microbiome of a group of 53 healthy subjects. A profound view was obtained of the interpersonal, intrapersonal and temporal diversity of the human axillary microbiota. Denaturing gradient gel electrophoresis (DGGE) and next generation sequencing on 16S rRNA gene region were combined and used as extent to each other. Two important clusters were characterized, where *Staphylococcus* and *Corynebacterium* species were the abundant species. Females predominantly clustered within the *Staphylococcus* cluster (87%, n = 17), whereas males clustered more in the *Corynebacterium* cluster (39%, n = 36). The axillary microbiota was unique to each individual. Left-right asymmetry occurred in about half of the human population. For the first time, an elaborate study was performed on the dynamics of the axillary microbiome. A relatively stable axillary microbiome was noticed, although a few subjects evolved towards another stable community. The deodorant usage had a proportional linear influence on the species diversity of the axillary microbiome.

## Introduction

The human skin harbors multiple niches, each being specified by a unique microbial community. Marples [1] compared the ecology of the skin with that of the earth: “the forearm is the desert, the scalps are the cool woods and the armpit is the tropical rainforest”. Certain skin niches are indeed more similar to those skin niches on another person than to any other skin niche on the same person [2]. The skin is, nevertheless, in constant interaction with microorganisms from the environment, as it is the most outer part of the body. Especially the dry areas, such as forearm, hand and buttock, contain a very diverse community with few interpersonal similarities [3], [4], [5]. The skin is, therefore, considered to preserve a complex microbial ecology, containing persistent resident bacteria, short-term resident bacteria and transient bacteria [6].

The elucidation of the human skin microbiome is of particular interest in studying disease states and the formation of body odor in, for instance, the axillae. The human axillae are covered by squames, hair shafts and follicles, eccrine, apocrine, sebaceous and, from puberty on, also apoeccrine glands [7], [8]. The armpits are characterized by warm, moist and nutritionally rich conditions. Salts, proteins, squalene, sterols, sterol esters, wax esters, a wide range of lipids and fatty acids are secreted, thus sustaining one of the highest densities of microorganisms on the body surface [9], [10]. Nonetheless the increasing use of deodorants and products alike, the axillae would be little vulnerable to environmental microbiota, due to its encompassed shape [11].

For decades, culture-dependent techniques have been the standard for characterizing the axillary microbial community. *Corynebacterium*, *Micrococcus*, *Staphylococcus*, *Propionibacterium*, and *Brevibacterium* have been regularly cultivated [11], [12]. These techniques form a sound basis but misrepresent the true bacterial diversity of a complex community as such [3]. Molecular methods have recently enhanced our knowledge of the diversity and ecology of the axillary microbiota, making use of DNA-based techniques, focusing on the microbial structure, as well as RNA-based techniques, focusing on the microbe's activity [13], [14]. High-throughput sequencing is becoming the new standard to characterize bacterial communities, including that of the human skin [15]. These molecular approaches in axillary microbiology are, nevertheless, in their initial phase. There is still a lack of knowledge regarding community composition, community structure (species richness and evenness), community dynamics (change over time), community variability (interpersonal and intrapersonal), the factors driving this variability and its relationship with disease states and body odor production.

This study conducted an elaborate molecular-based study in order to elucidate the axillary microbiome, its diversity and its dynamics. For the first time, a group of 53 subjects (17 females and 36 males) were sampled and analyzed using denaturing gradient gel electrophoresis (DGGE). Next-generation sequencing was used as a high-throughput molecular approach to identify and characterize the microbial communities of 9 specific individual axillary samples. The subjects were all working and living in Belgium, with 70% Belgians, 16% other EU citizens, 10% Asians and 4% South-Americans. Both axillae were sampled to determine the inter- and intrapersonal diversity. For the first time, the dynamics was studied over a period of nine months (19 subjects in total, with 7 subjects being sampled on regular successive time points). The subjects were asked for their daily hygiene habits and their deodorant usage in order to identify correlations.

## Results

### DGGE results indicate two main clusters

**Figure 1:**
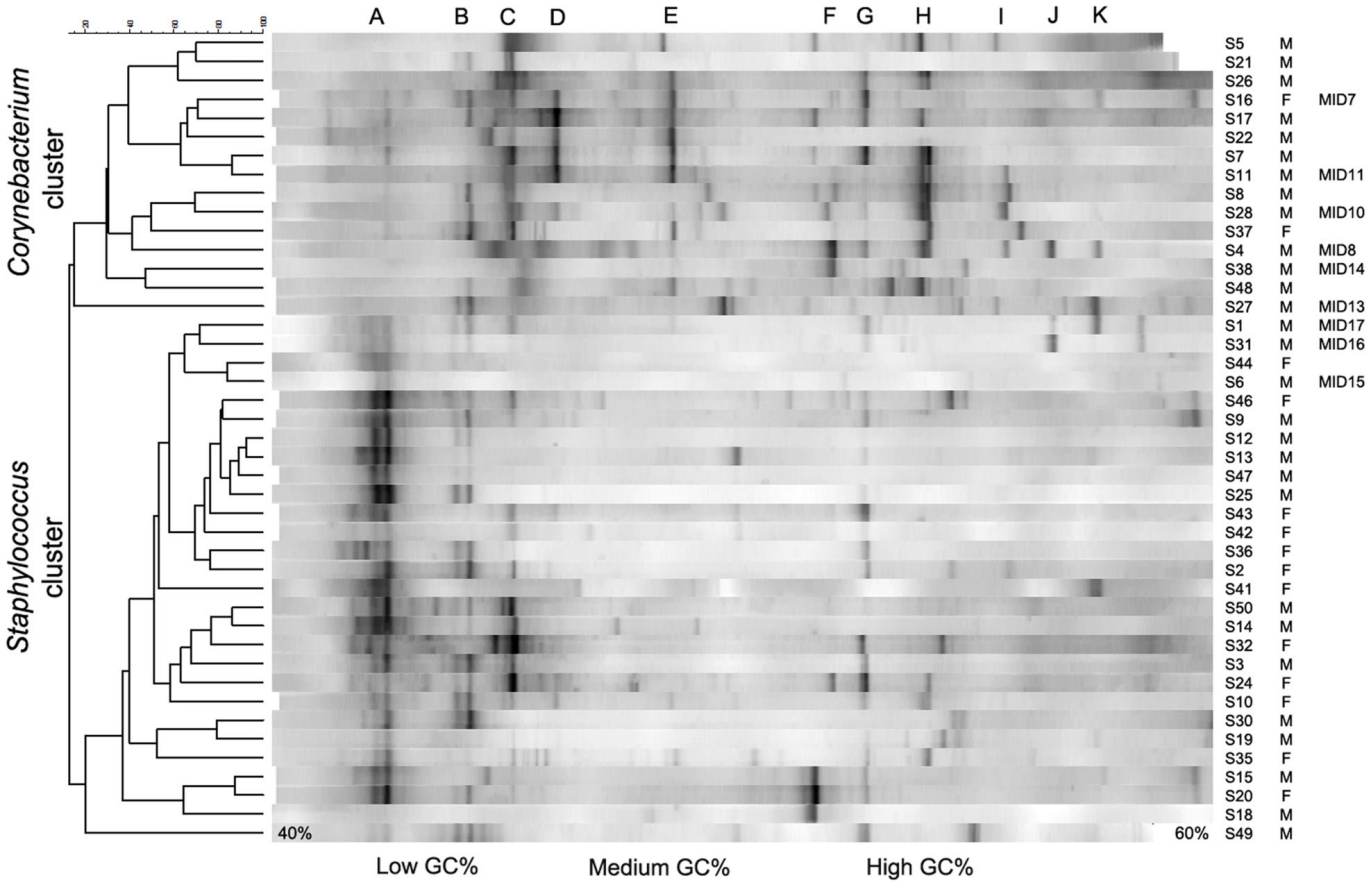
Clustering of individual axillary samples analyzed by means of DGGE, where 69% of the subjects clustered into the *Staphylococcus* cluster and 31% into the *Corynebacterium* cluster. Right: Subject indices from S1 till S53 (not all subjects were shown); gender of the subject; pyrosequenced samples indicated with MID (multiplex identifiers). Above: Identified band: bands A were identified as *Staphylococcus epidermidis* (100% identity), bands B were identified as *Staphylococcus* spp. (99% identity), bands C were identified as *Staphylococcus hominis* (100% identity), bands D and E were identified as *Proteobacteria* (from pyrosequencing results), band G was identified as *Corynebacterium* spp. (99% identity), bands H were identified as *Corynebacterium* spp. (99% identity), and bands F, I, J and K were identified as *Corynebacterium* spp. (from pyrosequencing results). Left: Clustering of the samples, based on Pearson correlation and unweighted pair group with mathematical averages dendrogram method. Under: indication of GC% of the bacterial bands. *Firmicutes* have a low GC%, and bands are generally situated left on the gel; *Actinobacteria* have a high GC%, with bands situated generally on the right side of the gel.

The individual left axillary samples of the 53 subjects were randomly loaded on a DGGE-gel. Two general clusters were formed. No better clustering could be achieved than the two main clusters. Clustering was performed in an unrooted way, independent from the further hypothesis testing. 61% of the subjects clustered in a group with *Staphylococcus* spp. as dominant species (hereafter mentioned as “*Staphylococcus* cluster”) (Figure 1). The main species of this cluster was *Staphylococcus epidermidis*, with prevalence of another *Staphylococcus* spp. and *Staphylococcus hominis*, all located within the higher denaturation area. These species belong to the *Firmicutes* phylum, known to contain a low GC%. The other axillary samples of the subjects (39%) formed a cluster with *Corynebacterium* spp., *Proteobacteria* and *Staphylococcus hominis*, identified as the main species. In this cluster, it seemed that these bacteria could grow into abundant quantities when *Staphylococcus epidermidis* was not present in dominant quantities. As *Corynebacterium* spp. was mainly present, this cluster was named “*Corynebacterium* cluster”. Identification of the species was realized through sequencing of the bacterial isolates (Table S2 in File S1) and pyrosequencing of bacterial communities.

### Diversity indices according DGGE results

The overall richness was estimated by means of range-weighted richness (Rr) and amounted about 9±7, which is rather low compared to other ecosystems [16]. The Rr of the *Corynebacterium* cluster and the *Staphylococcus* cluster amounted 10±7 and 7±6, respectively. A high variability was typified for the axillary microbiome across individuals. In order to describe the degree of evenness, the community organization (Co) was calculated, with an average of 12±4. The Co of both clusters were comparable, however, when looking at the samples with low richness (Rr<5) (low richness community was typified with a high abundance of *Staphylococcus epidermidis*), the Co of the *Staphylococcus* cluster was slightly higher than the *Corynebacterium* cluster, with a Co of 13±5 and 10±3, respectively. Table S5 in File S1 summarizes the diversity indices used to analyze the individual axillary samples.

### Temporal axillary diversity

**Figure 2:**
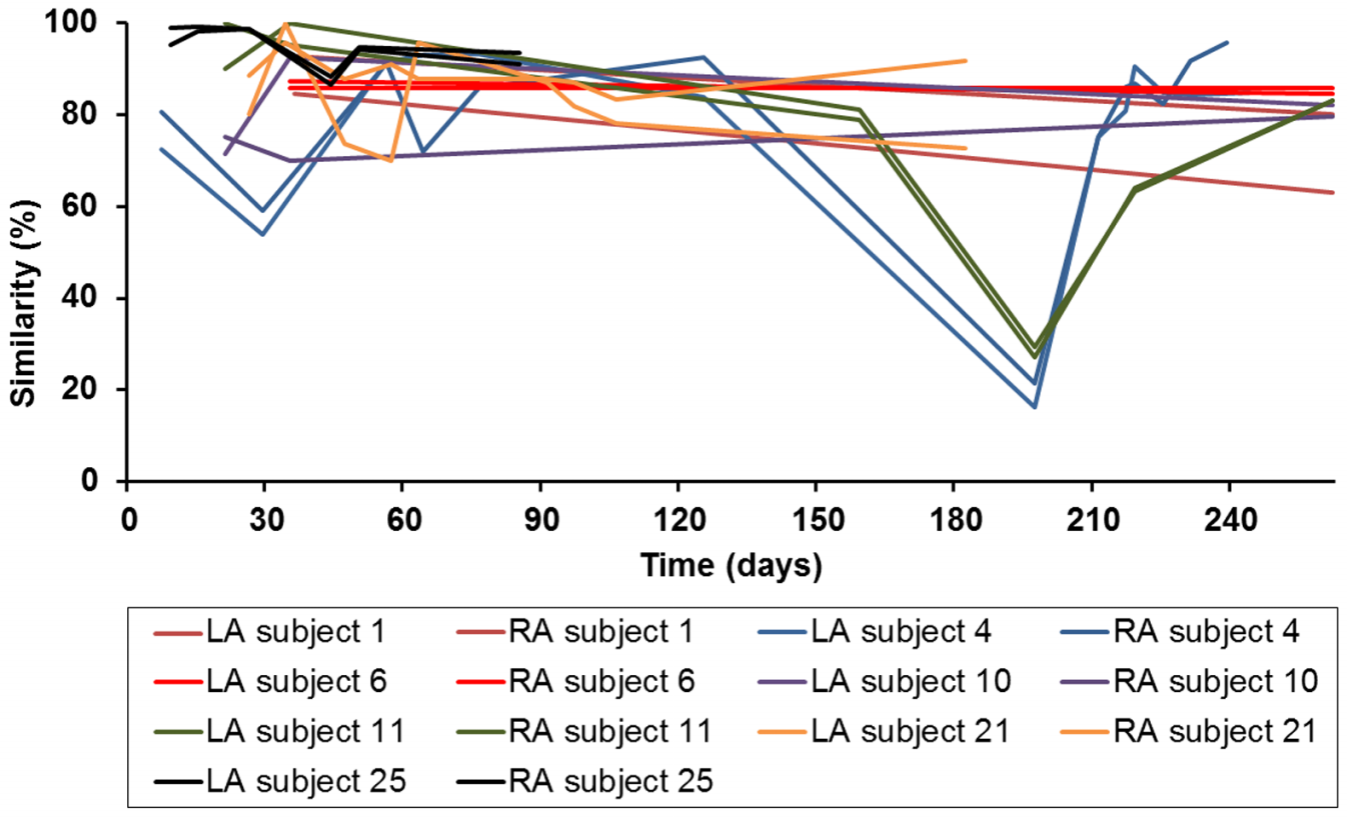
Dynamics (moving window analysis) of 7 subjects of the DGGE results. LA = left axilla; RA = right axilla. Left axis indicates the similarity (based on Pearson correlation) of the axillary sample compared to the previous axillary sample. The higher the curve, the more similar the samples. The axillary microbiome was relatively constant throughout time, even on a longer timescale (9 months). Two followed-up subjects experienced an community shift from one cluster to the other, after which the microbiome again was stable (subject 4 and 11).

The dynamics of the axillary microbiome was examined with DGGE by sampling 19 subjects throughout time, of whom 7 subjects were sampled on a more regular basis (represented in Figure 2). The majority of the subjects displayed relatively constant DGGE patterns, even on a longer time scale (9 months). Except for subject 11, all axillary samples continuously clustered within the same group (both cluster types occurred). The average dynamic similarity of the 19 subjects was quite high and amounted 81±11% (based on Pearson correlation), with 79±13% and 83±9% dynamic similarity of the *Corynebacterium* cluster and the *Staphylococcus* cluster, respectively. Two subjects experienced a microbial community shift, with a sudden similarity lower than 50%. These subjects axillary microbiomes evolved towards another stable community (subject 4 and 11 in Figure 2). Both subjects initially clustered in the *Corynebacterium* cluster. One subject went from the *Corynebacterium* cluster to the *Staphylococcus* cluster (subject 11). According to the questionnaire, this person had another partner, had been on holidays to warmer regions, and changed the deodorant type and quantity of use during the sampling period. The other subject stayed within the same armpit cluster, but the abundances altered from *Corynebacterium* species to *Proteobacteria*, whereas the *Corynebacterium* spp. were still present in lower abundances (subject 4). According the subject's questionnaire, the season changed and this person started with more heavy physical labor in another environment during the sampling period.

### Inter- and intrapersonal axillary diversity

A high interpersonal diversity was noticeable in the axillary vault, with an average similarity of 31±27%. When looking within one cluster, the similarities were significantly higher (p<0.0001) for the *Staphylococcus* cluster, with 55±21% similarity, in relation to 39±21% of the *Corynebacterium* cluster (heatmap Figure S1 in File S1). The lower similarities were due to the more diverse bacterial profile of the latter cluster. The clustering did not rely on the age, deodorant usage, shaving, hygiene habits or geographical differences. The subjects average deodorant usage amounted 6.6 times per week. No correlation was found between gender and deodorant use or cluster and deodorant use. The gender had a considerable influence with females predominantly clustering in the *Staphylococcus* cluster (87% of the females). In most of the cases, males clustered in the *Corynebacterium* cluster (39% of the males).

The similarity between left and right axillary bacterial community was quite high with an average of 87±11% (Table S8 in File S1). About 49% of the axillary samples showed –mostly minimal– differences in band pattern between left and right axilla. This left-right dissimilarity was seen in *Staphylococcus* as well as *Corynebacterium* cluster. The other 51% had the same bacterial community in both axillae (similarities of 78±14% and 95±4%, respectively). When looking at the samples taken on consecutive times of one subject, the left-right dissimilarity or similarity was not always consistent. The intrapersonal variability of the *Corynebacterium* cluster amounted an average similarity of 84±13% compared to 89±10% of the *Staphylococcus* cluster.

### Pyrosequencing results confirms two armpit clusters

**Figure 3:**
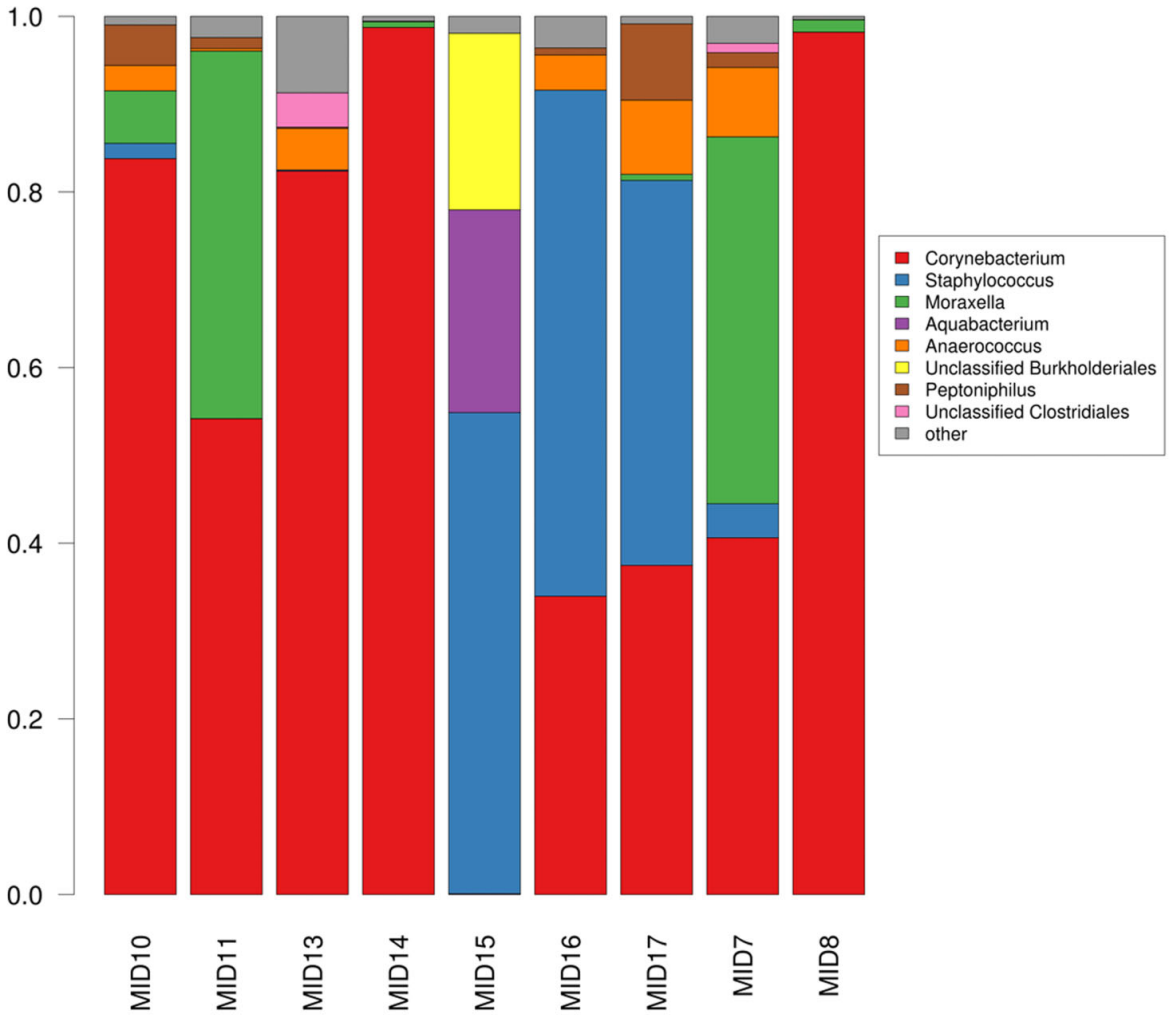
Stacked bar sample-wise taxonomic distribution of the sequences on genus level of the nine pyrosequenced axillary samples. MID7 is a sample of a female person (34 y, S16) using deodorant 24 times per week; MID8 is a sample of a male person (24 y, S4) using no deodorant; MID10 is a sample of a male person (24 y, S28) using deodorant 7 times per week; MID11 is a sample of a male person (27 y, S11) using deodorant 5 times per week; MID13 is a sample of a male person (27 y, S27) using deodorant 3 times per week; MID14 is a sample of a male person (25 y, S38) using deodorant 3 times per week; MID15 is a sample of a male person (23 y, S6) using deodorant 7 times per week; MID16 is a sample of a male person (29 y, S31) using deodorant 10 times per week; MID17 is a sample of a male person (35 y, S1) using deodorant 7 times per week. All subjects were Belgian. Additional subject metadata description can be found in Table S7 in File S1.

**Figure 4:**
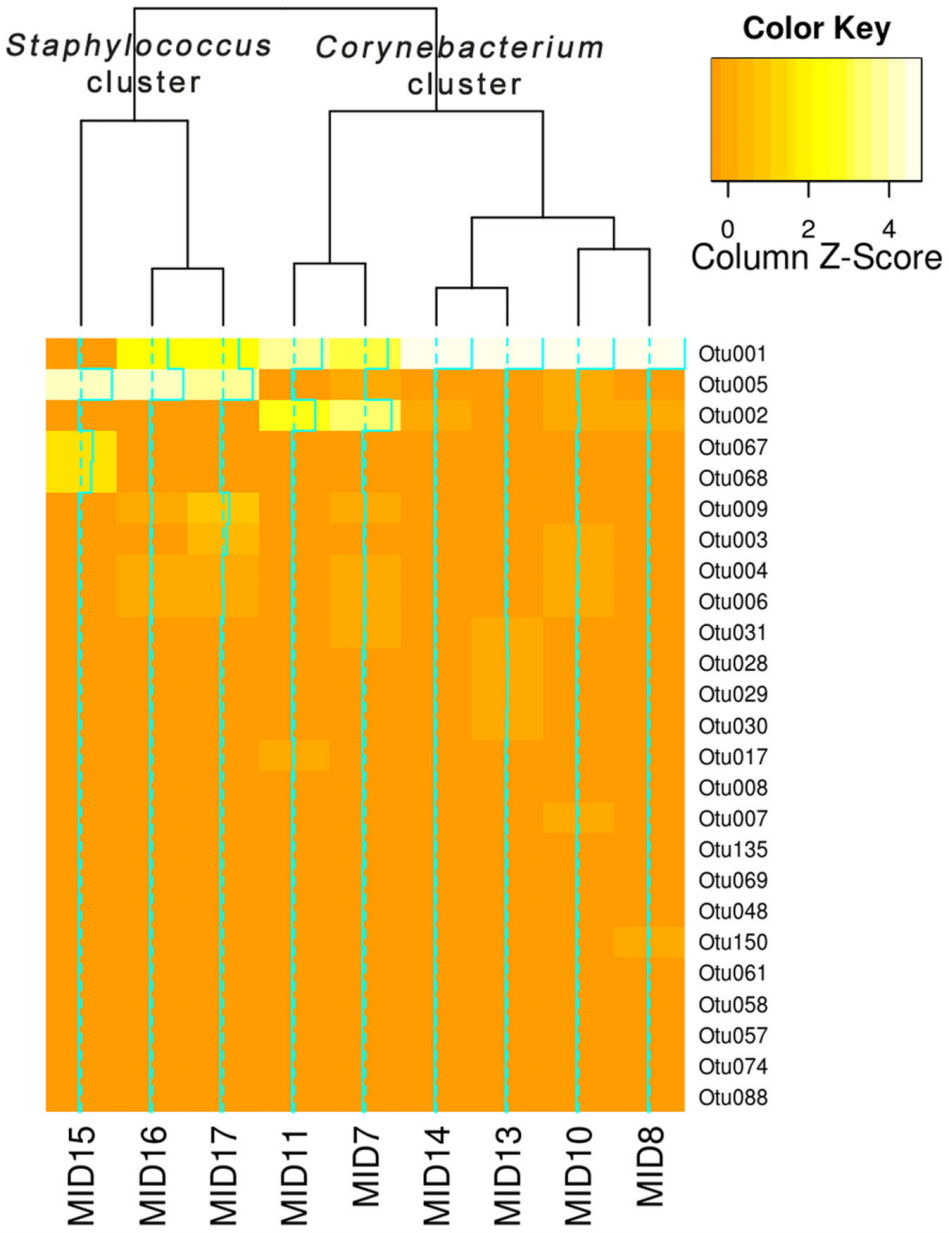
Heatmap and clustering of the top 25 OTUs of the pyrosequenced samples. Data was ranked according to the total count of the OTU among all the samples and samples were clustered using hierarchical clustering (complete linkage) and Bray-Curtis distance measures. OTU0001 = *Corynebacterium* spp., OTU0005 = *Staphylococcus* spp., OTU0002 = *Moraxellaceae* (*Proteobacteria*). Full OTU description can be found in Table S6 in File S1.

Based on DGGE, nine samples were chosen for 454 pyrosequencing analysis. Six samples were chosen from the *Corynebacterium* cluster and three samples from the *Staphylococcus* cluster. Especially those samples with bands for which we could not isolate the respective bacteria were selected for next-generation sequencing. Nine classified bacterial phyla were detected, with most sequences (99.59%) assigned to three phyla: *Actinobacteria* (59.7%), *Firmicutes* (23.2%), and *Proteobacteria* (16.7%). The *Bacteroidetes* phylum was represented in minimal quantities in eight of the nine subjects axillary samples (0.3% of total detected sequences). Of the 96 classified OTUs, two were associated with 76.7% of the sequences: *Corynebacterium* (59.5%; *Actinobacteria*), and *Staphylococcus* (17.2%; *Firmicutes*), as seen in Figure 3. Clustering of the nine pyrosequenced samples (indicated by MID on Figure 1) resulted into two clusters with *Staphylococcus* spp. and *Corynebacterium* spp. as abundant species (Figure 4; additional bar graphs in Figure S2 in File S1). Thorough descriptive beta diversity analysis showed significant differences between the two clusters (Figure S1A, S3; Table S4, all in File S1). As initially proposed by DGGE analysis, the six samples clustered into *Corynebacterium* cluster and the three samples in the *Staphylococcus* cluster.

### Axillary bacterial community characteristics according the pyrosequencing results

A total of 3263 16S gene sequences, based on 87646 sequence reads, were assigned into 159 unique OTUs, with a similarity threshold of 97%. OTUs belonging to the *Actinobacteria* phylum were predominantly identified as *Corynebacterium* spp. (up to 98.37% of the axillary microbiome of one subject). Other *Actinobacteria* members, often detected in axillary microbiomes by means of cultivation techniques, like *Micrococcus*, *Propionibacterium* and *Brevibacterium*, were detected in very low quantities in some of the samples, mostly 0.13%, 0.03%, and 0.02% of the total axillary microbiome of one subject, respectively. OTUs identified in the *Firmicutes* phylum were most commonly *Staphylococcus* species (up to 60.90% per subject). *Anaerococcus* and *Peptinophilus* genera were often detected, with a maximum up to 12.48% and 7.78% of one persons total community, respectively. The *Proteobacteria* phylum contained a wide range of genera. Mostly unclassified *Rhodocyclaceae* and *Acidovorax* spp. were found, next to *Paracoccus*, *Acinetobacter*, *Pseudomonas*, and *Escherichia* amongst others, present in minimal amounts. *Acidovorax* spp. was found in three out of nine axillary microbiomes, which is a species normally present in activated sludge, in soil and on deteriorating fruit and vegetables (amongst others), which one person was directly working with. *Lactobacillus* spp. were detected in very small quantities (0.01% and 0.25%) in two male axillae (MID16 and MID17).

### Diversity indices according pyrosequencing results

**Figure 5:**
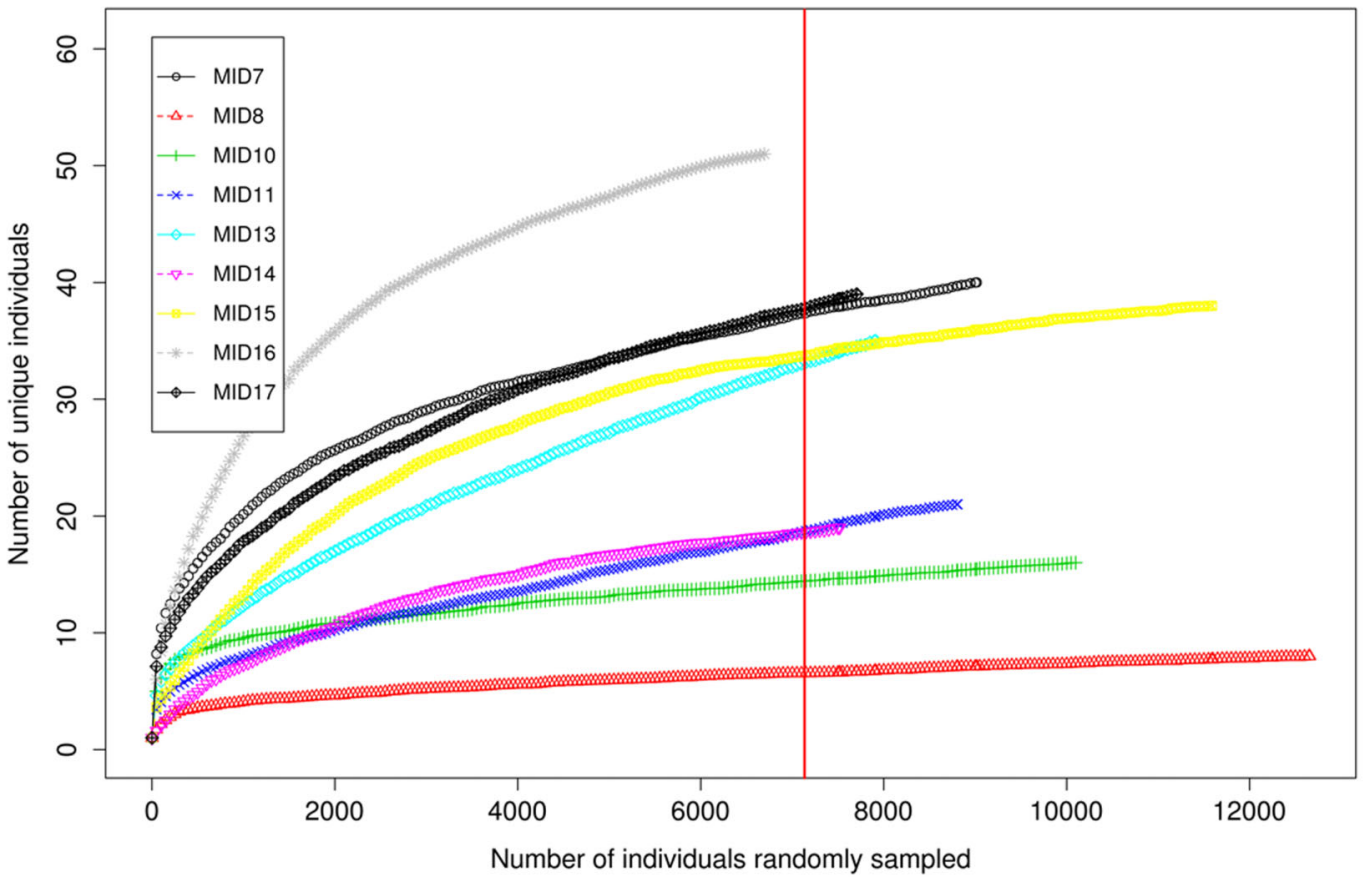
Rarefaction curve on the complete dataset of the pyrosequenced samples. The normalization cut-off was set on 7135 sequences, as indicated by the vertical red line.

**Figure 6:**
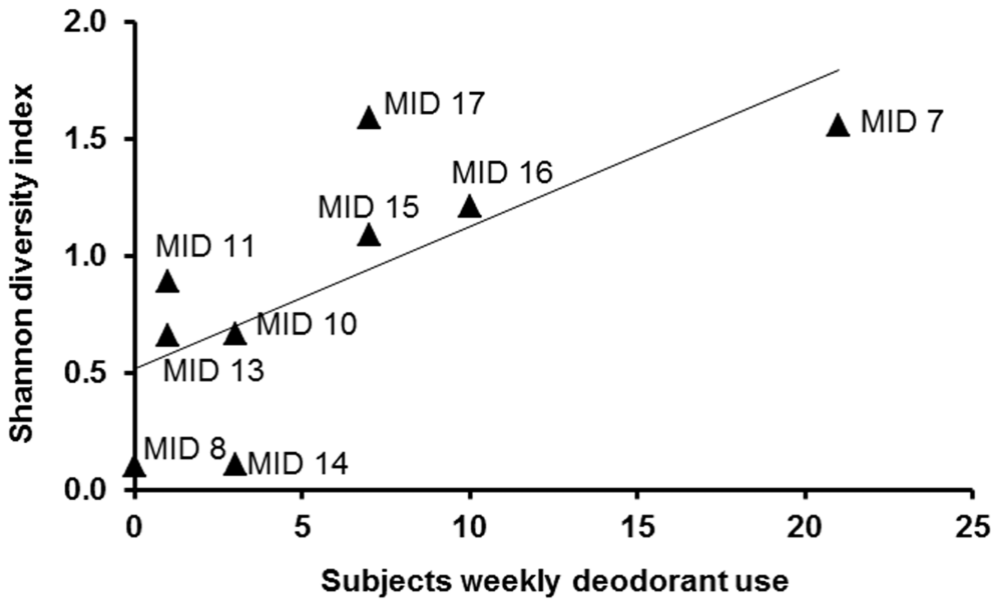
Shannon index for community diversity of all nine pyrosequenced samples in function of the subjects weekly deodorant use. The higher the subjects deodorant usage frequency, the higher the diversity of the microbial community. The line is an indication of the proportional correlation between deodorant use and species diversity.

The rarefaction curve is plotted as the number of unique sequences detected in function of the number of total detected sequences in the sample (Figure 5). It can be estimated that there were still 10 to 15 unique OTUs yet to be identified for some axillary samples. The observed and Chao1 estimated richness amounted 33±14 and 35±18.9 median OTUs per subject, respectively, with a broad distribution between the different subjects (Figure S4, Table S3, all in File S1). The most diverse bacterial sample included 66 phylotypes, and the least diverse included 6 phylotypes. A median of 4 phyla (range 3 to 7 phyla) and 16 families (range 8 to 36 families) per subject were identified. The Shannon diversity index was calculated in order to estimate the relative diversity (giving an idea of both richness and evenness) of the subjects axillary bacterial communities (Figure S5, Table S3, all in File S1). The index indicated an increased diversity for subjects with a higher deodorant usage frequency, as shown in Figure 6. The same proportional result was obtained when plotting the richness of the community in function of the amount of deodorant used per subject.

## Discussion

This study obtained a detailed characterization of the axillary microbiome of a large group of healthy subjects. Based on the pyrosequencing analysis, 99.59% of the sequences were assigned to only three phyla: *Actinobacteria* (59.7%), *Firmicutes* (23.2%) and *Proteobacteria* (16.7%). 76.7% of the sequences were associated with only two genera: *Corynebacterium* and *Staphylococcus*. *Staphylococcus* spp. and *Corynebacterium* spp. were typified as two of the most important species in the armpit. In this research, these species formed the basis for two clusters and were elaborately characterized. It should be appointed that, next to *Staphylococcus* spp. and *Corynebacterium* spp., also *Propionibacterium* spp. and *Proteobacteria* have been detected in the axillary vault in relatively abundant amounts [4], [5], [13], [17]. This research rather focused on the first two genera, as such, forming the basis for future research.

Both DGGE and 454 pyrosequencing analysis on 53 and 9 samples, respectively, indicated two distinct clusters. DGGE served as a quick and reliable screening of the microbial community and is still the most widely applied molecular technique for microbial profiling [18], [19], [20]. Pyrosequencing completed the picture with an in-depth analysis of the microbiome. According the DGGE results on 53 subjects, 61% clustered into the *Staphylococcus* cluster and 39% into the *Corynebacterium* cluster (Figure 1). The last group had a higher diversification of the community. *Corynebacterium* spp., *Proteobacteria*, *Firmicutes* and other bacteria were present in the same cluster. A microbial community shift was observed for a subject with this ecological pattern.

This research found that more than the half of the examined population carry an axillary bacterial community dominated by *Staphylococcus* spp. This is supported by culture-based [12], as well as molecular-based research [5], [13], [17]. The diversity of the *Staphylococcus* cluster was lower, with a higher interpersonal similarity. A more uneven community was observed for samples with a high abundance of *Staphylococcus epidermidis* present. These findings suggest that this species can have an inhibitory effect on other bacteria. *Staphylococcus* spp. are indeed known to cross-inhibit unrelated bacterial groups via quorum sensing [21]. The diversity of the axillary community was lower than that of the intestinal community. When observing the DGGE results, the Rr amounted 9 and 60, respectively, and the Co 12 and 40, respectively [22]. When observing the 454 pyrosequencing results, the median Chao1 richness estimator of the axillary skin was 35 compared to 135 OTUs in the intestinal community [23].

This work demonstrated the influence of the gender, where female subjects predominantly clustered in the *Staphylococcus* cluster (in 87% of the cases, for the DGGE results). Pyrosequencing results could not confirm this, due to the low amount of pyrosequenced samples. The gender influence is in correlation with Fierer *et al.* [24], who showed that *Corynebacterium* spp. reside better on a male hand skin, and Zeeuwen *et al.* [17], who found gender like differences on the upper buttock. Skin anatomical and physiological differences, as in hair growth, skin thickness, hormone, sweat and sebum production, form the basis for these microbial differences [25]. No specific clustering was found correlated with nationality, age, deodorant use, shaving or hygiene habits.

The nine armpit samples revealed 159 unique bacterial phylotypes, with a 3% cut-off, belonging to nine bacterial phyla. This is in correlation with what was found in other skin related studies [3], [14]. A median diversity per axillary sample was found of 35 phylotypes, with a minimum of 6 and a maximum of 66 phylotypes. Some armpits can consequently be a tenfold more diverse as others. This is a high interpersonal difference compared to the belly button, where a threefold was observed [26]. It is hypothesized that these differences are a consequence of the hygiene and cosmetics habits of the subjects.

The pyrosequencing results designated a proportional influence of the frequency of deodorant usage on the species richness and evenness (Figure 6). Although this research did not initially focus on this topic, a clear correlation could be seen between deodorant use and species diversity. As seen for the application of make-up on the forehead [27], a higher use of deodorants resulted in a higher diversity. It is suggested that deodorants, antiperspirants or perfumes, cause disruption of the bacterial community, accordingly, other bacteria can invade the community. The application of cosmetics on the axillary skin appears to open the skin niche, making it prone to colonization by bacteria from the surrounding environment.

The diversity of axillary microbiome increased as follows: interpersonal diversity>temporal diversity>intrapersonal diversity (based on the DGGE results). Regarding the interpersonal diversity, every individual possessed a unique axillary bacterial community. The impact of the deodorant usage and the direct environment can have an essential influence on the diversity, as determined in this research. Similar high interpersonal diversities were seen for the superficial skin and skin microbiome in general [3], [4], [5], [17]. The factors driving the diversity can be numerous. The individual axillary secretions, different hygiene habits and deodorant practice, amongst others, form the basis for the high variability in interpersonal similarities, richness and evenness. Within one cluster, the diversity was much lower, especially within the *Staphylococcus* cluster (Figure S1B in File S1). As such, the clustering around specific axillary species forms a characteristic property in the human axillary region.

The intrapersonal diversity was generally much lower than the interpersonal variation. Although a low intrapersonal diversity, about half of the analyzed axillary samples showed small differences in band pattern, suggesting left-right asymmetry in bacterial presence. The intrapersonal differences were seen on subjects using deodorants/antiperspirants as well as on subjects not using deodorants/antiperspirants. Similar intrapersonal diversities were seen in a previous study [13], however could not be seen by means of culturing [12]. The study of Egert *et al.* [13] showed that *Peptoniphilus* spp. were particularly active in the right armpit.

Few studies conducted the temporal microbial diversity of the skin, and were mainly based on no more than two intervals [28]. This study aimed on a better description of the temporal diversity of the human axillary microbiome, based on two to fourteen intervals over a period of nine months. A relative stable pattern was perceptible, even for longer periods, indicating a robust core axillary microbiome. Apparently, the axillary microbiome is more stable than the forearm skin and the palms [3], [29]. The axillary area is encompassed and typified by a warm, moist and nutritional rich environment, which could explain the stable microbiome. Nevertheless, two out of nineteen subjects experienced a microbial community shift, with a sudden rate of change higher than 50%, after which the microbiome was again stable. One of those subjects permanently changed from the *Corynebacterium* cluster to the *Staphylococcus* cluster. External factors were most probably the cause for these changes. Subjection to other environmental, nutritional and seasonal conditions could alter the axillary microbiome. The fact that such conditions can modify the microbiome implies that control of the axillary microbiome might be conceivable.

This study is a step forward in a better understanding of the healthy axillary microbiome and forms the baseline for further studies on the axillary microbiome in relation to disease states and body odor generation. Deodorants and antiperspirants clearly seem to influence the axillary microbiota, making further research on this topic necessary. A bacterial community shift from one to the other cluster was possible, making it plausible to gain control over the axillary clusters. As such, this could form a solution towards bromhidrosis and other cutaneous diseases.

## Methods

### Study design

Triplicate specimens were obtained from the left and right axilla of 53 subjects, with no history of dermatological disorders or other chronic medical disorders and with no current skin infections. A moistened (with sterile saline) sterile cotton swab was thoroughly swabbed in the axillary region for 15 s, upon which it was vigorously rotated in a reaction tube with 1.0 ml of sterile saline water to transfer the bacteria [30]. DNA extraction was adapted from Rodriguez-Lazaro *et al.* [31]. The 16S rRNA genes of all samples were amplified by PCR and loaded on DGGE, based on the protocol of Muyzer *et al.* [32]. 5 different bacterial isolates were obtained and sequenced for identification, making use of Sanger sequencing. The isolates were compared with the mixed DGGE profiles in order to identify the bacterial groups. 9 axillary samples were chosen, based on the DGGE profiles, and analyzed in-depth by means of 454 pyrosequencing. 19 of the 53 subjects were sampled more than once in a period of 9 months, in order to elucidate the dynamics. 12 of the 19 subjects were sampled again after 9 months. 7 of the 19 subjects were samples 3 to 14 times with regular intervals during the 9 months in order to obtain a more profound view of the dynamics. The axillary samples of the different subjects were compared in order to identify the interpersonal diversity. Left and right axillary samples of the same subject were compared in order to identify the intrapersonal diversity. The used primers, targeted hypervariable 16S rRNA gene regions and PCR programs of all methods are represented in Table S1 in File S1. Descriptive alpha and beta diversity statistics, cluster analysis and hypothesis testing were performed. The alpha diversity indices to analyze DGGE and pyrosequencing results are represented in Table S5 in File S1. Full description of M&M can be found in File S1.

### Ethics Statement

The study was approved by the Ghent University Ethical Committee with approval number B670201112035. All participants gave their written consent to participate in this study as well as consent to publish these case details.

## Supporting Information

File S1 **Online Supporting Information.**
(PDF)
