# Supplementary material for: Characterization of Staphylococcus and Corynebacterium Clusters in the Human Axillary Region
Source: PLoS One. 2013 Aug 12;8(8):e70538. doi: 10.1371/journal.pone.0070538 (PMC3741381; doi:10.1371/journal.pone.0070538)
Supplement: File S1 — Online Supporting Information. (PDF) [file pone.0070538.s001.pdf]

SUPPLEMENTARY ONLINE MATERIAL

Subjects

Triplicate specimens were obtained from the left and right axilla of 53 healthy subjects. The subjects had no history of dermatological disorders or other chronic medical disorders and had no current skin infections. The subjects were asked by the researcher about their skin health and if no antibiotics was used on the moment of sampling and in the months before sampling. Samples were collected over a period of one year from volunteers working in the area of Ghent (Belgium), with a temperate maritime climate by the North Sea and Atlantic Ocean. The subjects were asked to fill in a questionnaire to collect their metadata (Figure S7). 35 persons (70%) were Belgian, 8 persons (16%) were non-Belgian EU citizens (Greece (2), France, Portugal, Italy, Finland, Germany, The Netherlands) and 7 persons (14%) originated from Asia and South-America (Indonesia (3), Vietnam, India, Uruguay, Peru). The mean age was 29 years (range 21 to 65 years), with 32% (17 out of 53) females and 68% (36 out of 53) males. All were in good health and had not received any antibiotics for at least one month. No attempts were made to control the subjects diet, hygiene habits or deodorant/antiperspirant usage. No attempts were made to determine the odour profile of the subjects armpit. 94% of the subjects used a deodorant or antiperspirant, with an average of 6.6 times per week. All subjects gave their written and informed consent to this research.

Sampling

A moistened sterile cotton swab (Biolab) was thoroughly swabbed for 15 s in the axillary region to detach and absorb the microorganisms, upon which it was vigorously rotated for 15 s in a sterilized reaction tube filled with 1.0 ml of sterile saline water to transfer the bacteria [1]. The

subjects swabbed their total axillary region three times on the same areas of skin with, each time, a new sterile cotton swab. The subjects were thereby guided by the researcher, who informed about the exact location and during of sampling. The researcher handed the new sterile cotton swab, who were first moistened with sterile saline. The swab method was chosen as it is a convenient, non-invasive and relatively reproducible technique to take axillary samples from a large group of persons. As shown on the skin before [2], swabbing is a comparable technique to scraping when analyzing the microbial community and diversity. The bacterial samples were immediately frozen at -20°C for further DNA-extraction. Successive samples were taken from 19 subjects, with minimum one week in between sampling. To minimize the effect of washing and deodorant use, the samples were taken on the same hour of the day in the late afternoon. All subjects had normal working hours. Exclusion or normalization of deodorant use and washing habits was, nevertheless, impossible. Detailed subject metadata are represented in Table S7.

DNA extraction

Total DNA extraction was adapted from Rodriguez-Lazaro *et al.* [3]. Briefly, the bacterial sample, dissolved in 1.0 ml of saline water, was centrifuged (12 min, 13,000 rpm) to obtain a pellet, while the supernatant was discarded. The pellet was resuspended in 100 µL 6% Chelex-100 resin (BioRad, Munich, Germany) and incubated at 56°C for 20 min. The sample was then firmly vortexed and boiled at 100°C for 8 min. Subsequently, the sample was mixed and cooled for 5 min on ice. Next, a centrifugation step (10 min, 13,000 rpm) was performed. The supernatant containing the DNA was removed and stored at -20°C until further analysis. The removal of PCR inhibitors and metal ions was accomplished by means of Chelex-100.

PCR for DGGE

The 16S rRNA genes for all bacteria were amplified by PCR using the forward primer P338F and the reverse primer P518R [4,5]. A GCclamp of 40 bp [4,5] was added to the reverse primer. The used primers, targeted hypervariable 16S rRNA gene region and PCR program are represented in Table S1 A and B. Positive and negative controls were added to the sample pool in order to check the PCR reaction. All sample pools with false positive or false negative results were discarded. Amplicons were analyzed by electrophoresis in 1.5% (wt/vol) agarose gels stained with ethidium bromide. A clearly visible band was present, which suggested that no aspecific amplification occurred. The PCR for DGGE was repeated several times, using different cycle conditions and starting DNA quantities, to counteract possible PCR differences, where no bias was seen on DGGE.

DGGE analysis

DGGE (Denaturing Gradient Gel Electrophoresis) based on the protocol of Muyzer *et al.* [4] was performed using the INGENYphorU System (Ingeny International BV, The Netherlands). PCR fragments were loaded onto 8% (w/v) polyacrylamide gels in 1 × TAE buffer (20 mM Tris, 10 mM acetate, 0.5 mM EDTA pH 7.4). To process and compare the different gels, a homemade marker of different PCR fragments was loaded on each gel [6]. The polyacrylamide gels were made with denaturing gradients ranging from 40% to 60% (where 100% denaturant contains 7 M urea and 40% formamide). The electrophoresis was run for 16 hours at 60°C and 120V. Staining and analysis of the gels was performed as described previously [7]. The normalization and analysis of DGGE gel patterns was done with the BioNumerics software 5.10 (Applied Maths, Sint-Martens-Latem, Belgium). During this processing, the different lanes were defined,

background was subtracted, differences in the intensity of the lanes were compensated during normalization and bands and band classes were detected. Clustering was done with Pearson correlation and the unweighted pair group with mathematical averages (UPGMA) dendrogram method. Relevant and non-relevant clusters were separated by the statistical Cluster Cutoff method (BioNumerics Manual 5.10). Similarities and abundances were extracted from the software and statistical analysis was performed using SPSS version 19. Significant cut-off values were indicated in the paper. Triplicate specimens of each sample were loaded on DGGE-gel, pooled and checked. No differences were seen.

Denaturing Gradient Gel Electrophoresis (DGGE) diversity indices

The range-weighted richness (Rr) is a DGGE specific range of values which indicate the richness and genetic diversity of species within the bacterial community. It is correlated with the distribution of the bands in the DGGE pattern and the percentage denaturant gradient of the gel needed to represent the sample's total diversity (within the limits of the technique). This is mathematically expressed as $Rr=N^2 \times D_g$, where N represents the total number of bands in the pattern, and D_g the denaturant gradient comprised between the first and the last band of the pattern [8]. The community organization (Co) describes the species abundance distribution in the microbial community and is calculated as the Gini coefficient times 100 [9]. The Gini coefficient (ranging from zero to one) is a single value that describes a specific degree of evenness measuring the normalized area between a given Pareto–Lorenz (PL) curve and the perfect evenness line. The higher the Gini coefficient, the more uneven a community is. The PL evenness distribution curve was constructed based on the DGGE profiles as previously described [8,10]. The community dynamics (Dy) was studied computing the moving window analysis

(MWA) plot of consecutive DGGE profiles of the same subject. The microbial community rate of change was conducted using the UPGMA and distance matrices of each DGGE based on the Pearson correlation similarity coefficient to cluster the succeeding samples [8].

Sanger sequencing

The 16S rRNA genes of five isolated pure strains, plated on blood agar plates, was amplified by PCR using the forward primer P63F and the reverse primer P1387R [11]. The PCR program was performed and checked as described in Table S1B. Sanger sequencing was performed on the 16S rRNA amplicons and aligned and compared with sequences from the NCBI database. The closest match of each isolate was identified. Afterwards, sequences of all strains were submitted to GenBank and the submission numbers are presented in Table S2.

Pyrosequencing

Amplicon pyrosequencing was performed on the total DNA extracted from nine specific individual samples. Barcoded amplicons were prepared with the primers 530F-mod [12] and 1061R [13] amplifying a 562 bp DNA fragment flanking the V4, V5 and V6 regions of the 16S rRNA gene [14], extended as amplicon fusion primers with respective primer L adaptor, key sequence and multiplex identifiers (MID) on the forward primer. Amplicons were generated by using FastStart High Fidelity Taq DNA Polymerase kit (Roche) under the conditions mentioned in Table S1B. Amplicons were purified with the High Pure PCR Product Purification Kit (Roche) and pooled as specified by the manufacturer. The purity and quality of the PCR products was verified on agarose gel. Emulsion PCR, emulsion breaking and sequencing were performed applying the GS FLX Titanium chemistry protocols and using a 454 GS FLX pyrosequencer

(Roche) as recommended by the manufacturer. For this study, nine amplicons were sequenced in a pool of 17 mixed amplicons on 1/4th of an FLX picotitre plate. Quality filtering of the pyrosequencing reads was performed using the automatic amplicon pipeline of the GS Run Processor (Roche), with a modification of the valley filter (vfScanAll- Flows false instead of TiOnly) to extract sequences. The raw flowgrams were processed and analyzed in an in-house Mothur [15] (http://www.mothur.org, version 1.24.1) and R (version 2.15)/Sweave pipeline. Sequencing error was reduced using the Mothur implementation of the SeqNoise algorithm [16], alignment with the SILVA 16S reference alignment [17] was performed and sequences were trimmed to overlap in the same alignment space. Chimeric sequences were removed using Uchime [18]. A Bayesian classifier was used with version 7 of the RDP training set [19] to classify the sequences. 87,646 bacterial 16S gene sequences were detected. 3,263 unique operational taxonomic units (OTUs) were assessed to a 97% confident p-value into 159 OTUs, with subsequent classification to unique classes into 96 classified OTUs. To allow for comparative community distribution analysis the samples were normalized at 7135 sequences, according to the sample with the lowest number of sequences. A .fastq file was created and submitted to NCBI with SRA study accession: SRP023149. Descriptive alpha and beta diversity statistics were calculated using mothur and visualized with R.

Alpha diversity analysis

The alpha diversity was calculated to characterize the diversity of one individual axillary sample. Figure S6 displays the range-weighted richness and the community organization of 43 individual DGGE samples. For the pyrosequenced samples, the analysis was performed on both the subsampled (with normalization at 7135 sequenses) and complete (without normalization)

dataset. The Shannon diversity index and the Chao 1 richness estimator were calculated and plotted in Figure S5 and S4, respectively. To assess the completeness of sampling also a rarefaction analysis was performed, and plotted in Figure 5. Detailed alpha diversity characteristics are displayed in Table S3. The Shannon diversity index, Chao1 richness estimator and observed richness of the axillary samples of the nine subjects were plotted in function of their weekly deodorant usage (Figure 6).

Beta diversity analysis

The beta diversity analysis was calculated to study the difference between the individual microbial axillary communities. A heatmap was generated in Figure 4 of the top 25 OTU's of the subsampled dataset with hierarchical complete linkage clustering based on Bray-Curtis similarities. Next, a heatmap of the Yue and Clayton θ dissimilarity index of the pairwise comparison of the pyrosequenced samples is shown in Figure S1A. Figure S1B represents a heatmap of the similarities of the different samples analyzed by DGGE.

Hypothesis testing

Additional hypothesis tests were conducted in order to identify whether the two cluster are dissimilar or not. The null-hypothesis (H_0) is that the community structures are similar. The parsimony method (aka P-test) is a generic test that describes whether two or more communities have the same structure. The results of the parsimony testing procedure are displayed in Table S4A. A cluster dendrogram is figured in Figure S3A clustered by UPGMA with the Bray-Curtis index for community structure. Nonmetric multidimensional scaling (NMDS) is efficient at identifying underlying gradients and representing relationships based on various types of

distance measures. The NMDS plot is displayed in Figure S3B of the pyrosequenced samples, based on the abundance-based Jaccard distance measure. Using molecular variance (amova) it is tested whether the centers of the clouds of the axillary clusters are separated or not. In Table S4B, it is tested whether the observed cluster separation between the *Corynebacterium* and *Staphylococcus* clusters in the NMDS plots is statistically significant.

DGGE versus Sanger sequenced isolates versus 454 pyrosequencing

In this research, DGGE fingerprinting, Sanger sequencing of isolates and 454 pyrosequencing were successfully combined. DGGE is a relatively rapid method, well suited for mixed microbial communities and is an interesting technique to study the community dynamics and diversity. *Firmicutes* and *Actinobacteria* are important phyla on the skin, with very dissimilar GC content. *Firmicutes* are known to possess a low GC content, whereas *Actinobacteria* are known to have a high GC content. As such, DGGE is a very solid technique to differentiate amongst the two. The disadvantages of DGGE were resolved by combining with 454 pyrosequencing and sequencing of isolated bacteria. Although isolation is only possible for a subset of the skin bacteria, some bands on DGGE were identified by combining the DGGE pattern of the pure culture with the pattern of the mixed axillary culture. 454 pyrosequencing made identification on genus level and quantification possible, but was a less flexible and a rather slow method, due to the rigorous analytic and statistical work. Figure 1 presents the results of all 3 techniques combined, where clear cohesion of all the techniques was found.

SUPPLEMENTARY REFERENCES

1. Evans CA, Stevens RJ (1976) Differential quantitation of surface and subsurface bacteria of normal skin by combined use of cotton swab and scrub methods. Journal of Clinical Microbiology 3: 576-581.
2. Grice EA, Kong HH, Renaud G, Young AC, Bouffard GG, et al. (2008) A diversity profile of the human skin microbiota. Genome Research 18: 1043-1050.
3. Rodriguez-Lazaro D, Jofre A, Aymerich T, Hugas M, Pla M (2004) Rapid quantitative detection of Listeria monocytogenes in meat products by real-time PCR. Applied and Environmental Microbiology 70: 6299-6301.
4. Muyzer G, Dewaal EC, Uitterlinden AG (1993) Profiling of complex microbial-populations by denaturing gradient gel-electrophoresis analysis of polymerase chain reaction-amplified genes-coding for 16S ribosomal-RNA. Applied and Environmental Microbiology 59: 695-700.
5. Ovreas L, Forney L, Daae FL, Torsvik V (1997) Distribution of bacterioplankton in meromictic Lake Saelenvannet, as determined by denaturing gradient gel electrophoresis of PCR-amplified gene fragments coding for 16S rRNA. Applied and Environmental Microbiology 63: 3367-3373.
6. Boon N, De Windt W, Verstraete W, Top EM (2002) Evaluation of nested PCR-DGGE (denaturing gradient gel electrophoresis) with group-specific 16S rRNA primers for the analysis of bacterial communities from different wastewater treatment plants. Fems Microbiology Ecology 39: 101-112.
7. Boon N, Goris J, De Vos P, Verstraete W, Top EM (2000) Bioaugmentation of activated sludge by an indigenous 3-chloroaniline-degrading Comamonas testosteroni strain, I2gfp. Applied and Environmental Microbiology 66: 2906-2913.
8. Marzorati M, Wittebolle L, Boon N, Daffonchio D, Verstraete W (2008) How to get more out of molecular fingerprints: practical tools for microbial ecology. Environmental Microbiology 10: 1571-1581.
9. Read S, Marzorati M, Guimaraes BCM, Boon N (2011) Microbial Resource Management revisited: successful parameters and new concepts. Applied Microbiology and Biotechnology 90: 861-871.
10. Wittebolle L, Marzorati M, Clement L, Balloi A, Daffonchio D, et al. (2009) Initial community evenness favours functionality under selective stress. Nature 458: 623-626.
11. Lane DJ (1991) 16S/23S rRNA sequencing. In: Stackebrandt E, Goodfellow M, editors; Stackebrandt E, Goodfellow M, editors. Nucleic acid techniques in bacterial systematics. Chichester, United Kingdom: John Wiley & Sons: 115–175.
12. Dowd SE, Sun Y, Secor PR, Rhoads DD, Wolcott BM, et al. (2008) Survey of bacterial diversity in chronic wounds using Pyrosequencing, DGGE, and full ribosome shotgun sequencing. BMC Microbiology 8.
13. Andersson AF, Lindberg M, Jakobsson H, Backhed F, Nyren P, et al. (2008) Comparative Analysis of Human Gut Microbiota by Barcoded Pyrosequencing. Plos One 3.
14. Neefs JM, Vandepeer Y, Derijk P, Chapelle S, Dewachter R (1993) Compilation of small ribosomal-subunit RNA structures. Nucleic Acids Research 21: 3025-3049.
15. Schloss PD, Westcott SL, Ryabin T, Hall JR, Hartmann M, et al. (2009) Introducing mothur: Open-Source, Platform-Independent, Community-Supported Software for Describing and Comparing Microbial Communities. Applied and Environmental Microbiology 75: 7537-

7541.
16. Quince C, Lanzen A, Davenport RJ, Turnbaugh PJ (2011) Removing Noise From Pyrosequenced Amplicons. Bmc Bioinformatics 12.
17. Pruesse E, Quast C, Knittel K, Fuchs BM, Ludwig WG, et al. (2007) SILVA: a comprehensive online resource for quality checked and aligned ribosomal RNA sequence data compatible with ARB. Nucleic Acids Research 35: 7188-7196.
18. Edgar RC, Haas BJ, Clemente JC, Quince C, Knight R (2011) UCHIME improves sensitivity and speed of chimera detection. Bioinformatics 27: 2194-2200.
19. Cole JR, Wang Q, Cardenas E, Fish J, Chai B, et al. (2009) The Ribosomal Database Project: improved alignments and new tools for rRNA analysis. Nucleic Acids Research 37: D141-D145.

Callewaert *et al*., Figure S1. Heatmap to assess the interpersonal diversity. (A) Heatmap of the Yue and Clayton θ dissimilarity $(1 - D_{\theta_{YC}})$ index of the pairwise comparison of 9 pyrosequenced sampled community structures to assess the interpersonal diversity. The higher the index (red), the more similar the communities; (B) Heatmap of the DGGE similarities of 43 sampled community structures. The higher the index (red), the more similar the communities. The nine pyrosequenced samples are indicated with MID (multiplex identifiers).

A

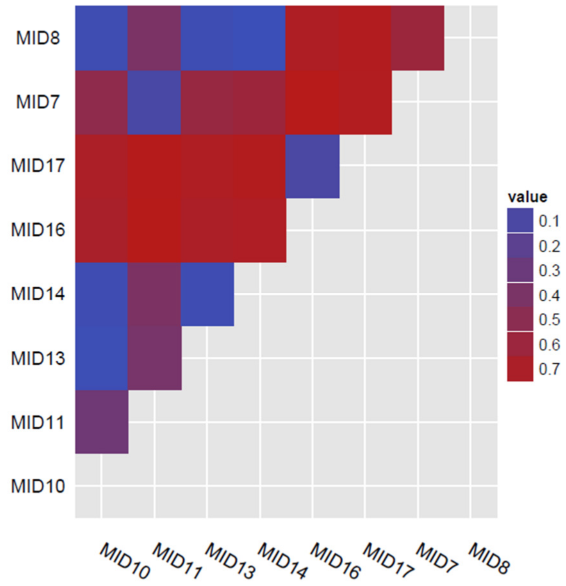

B

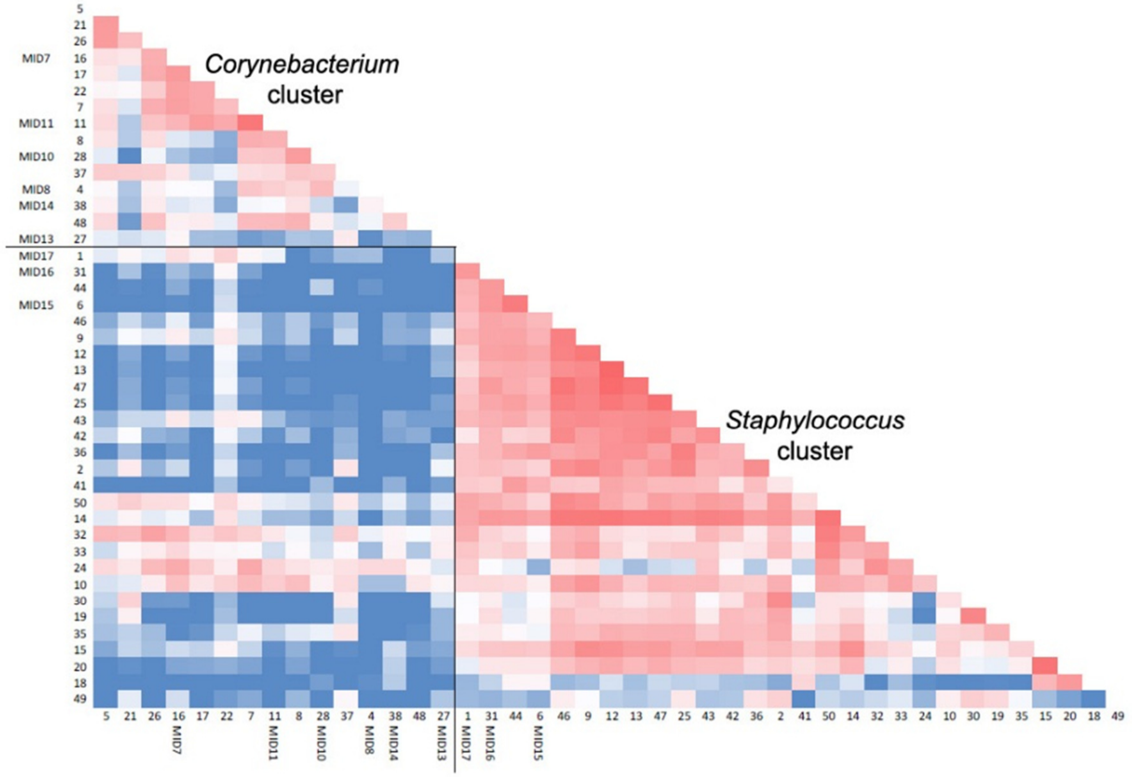

Callewaert *et al.*, Figure S2. Stacked bar sample-wise taxonomic distribution of the sequences (A) on the order level; (B) on the class level; (C) on the phylum level.

A

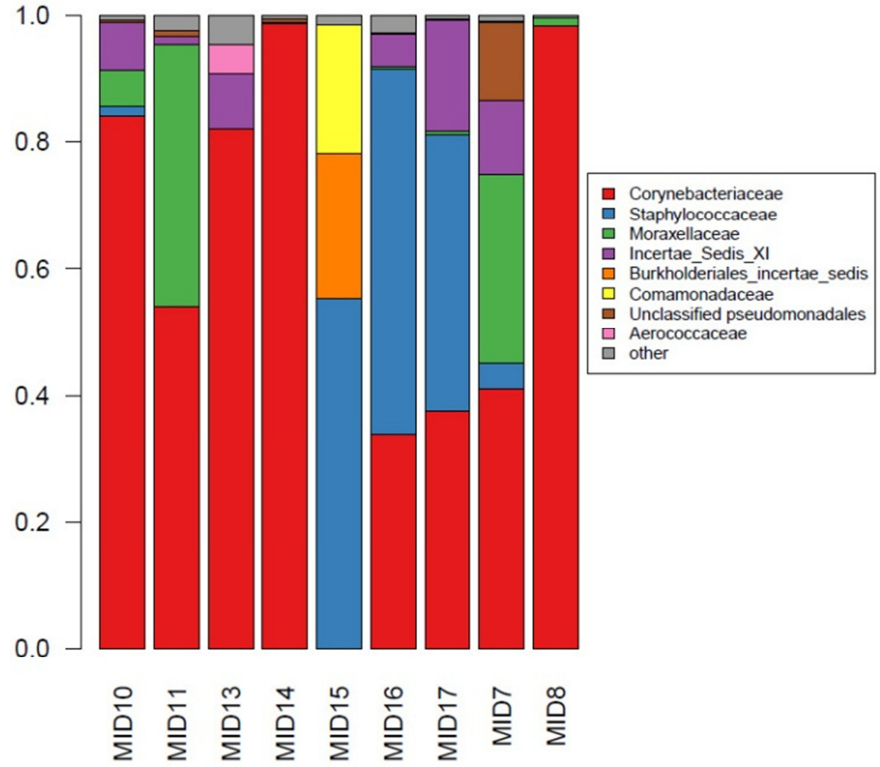

B

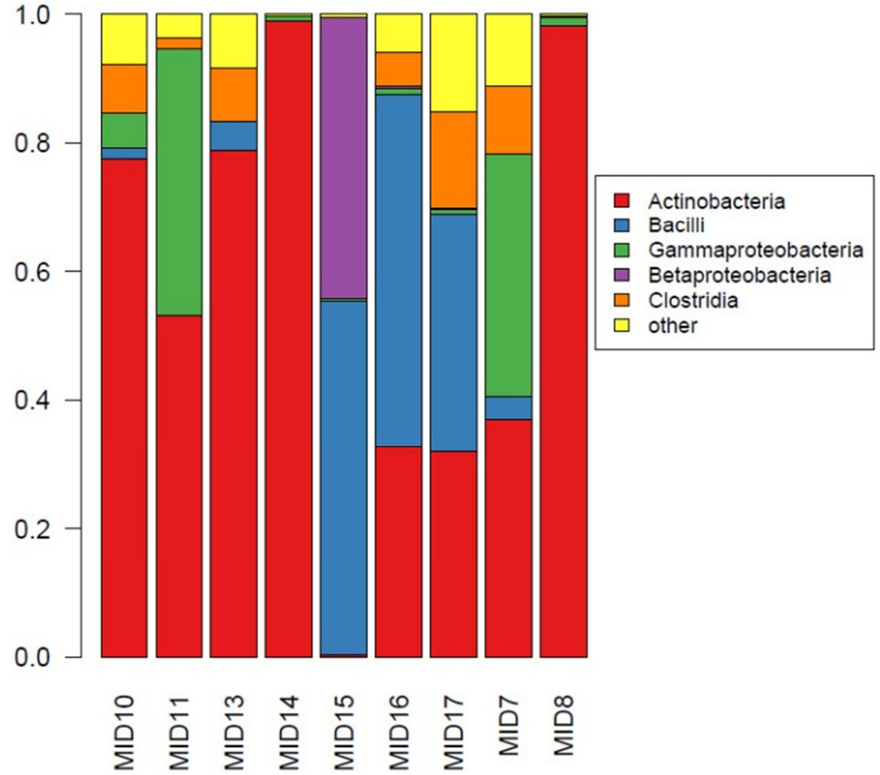

Callewaert *et al*., Figure S2. Stacked bar sample-wise taxonomic distribution of the sequences (A) on the order level; (B) on the class level; (C) on the phylum level.

C

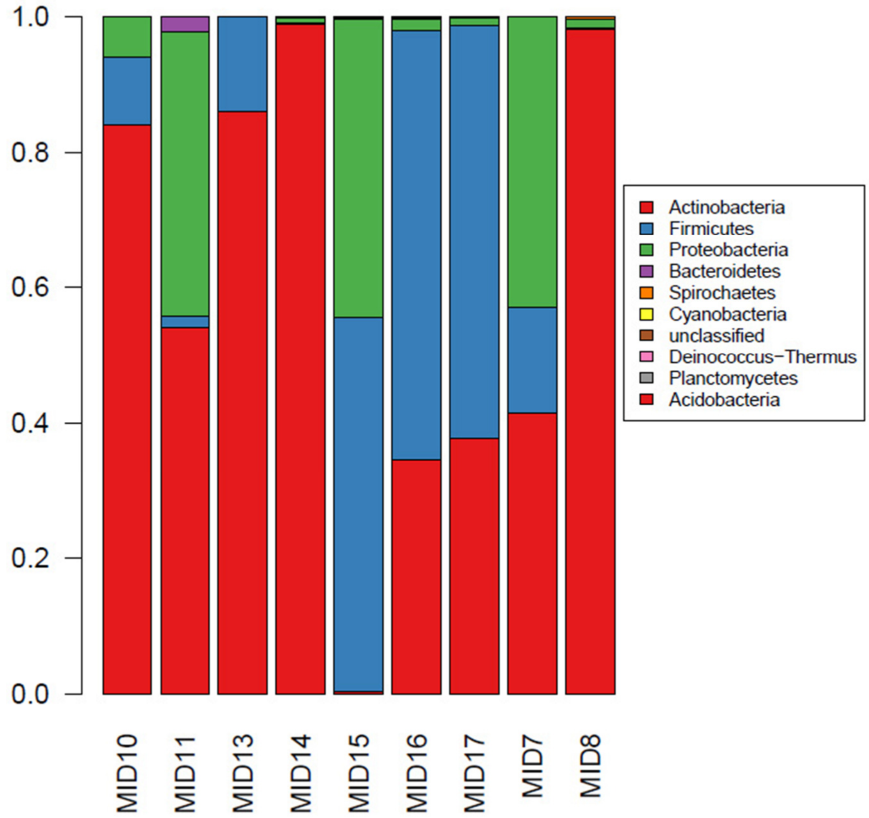

Callewaert *et al*., Figure S3. (A) Cluster dendrogram by UPGMA with the Bray-Curtis index for community structure. (B) NMDS plot based on the abundance-based Jaccard distance measure. Red sample names are from the Corynebacterium cluster, blue sample names Staphylococcus cluster.

A

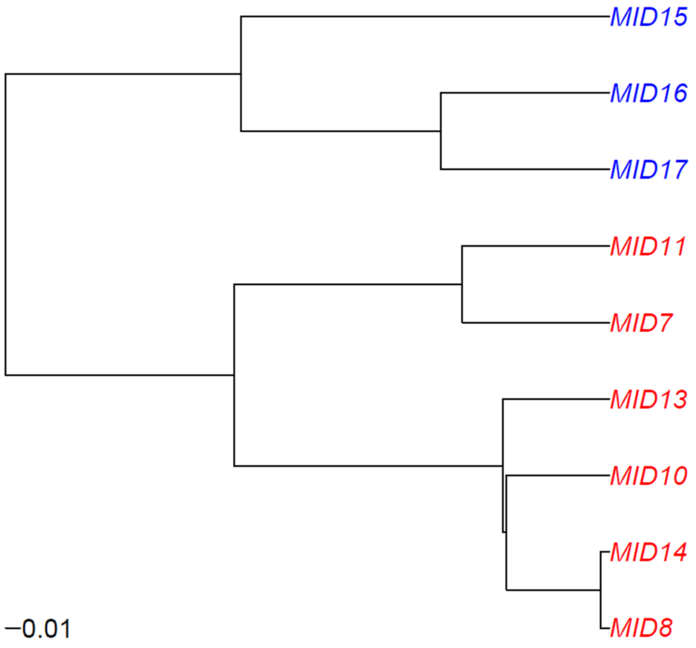

B

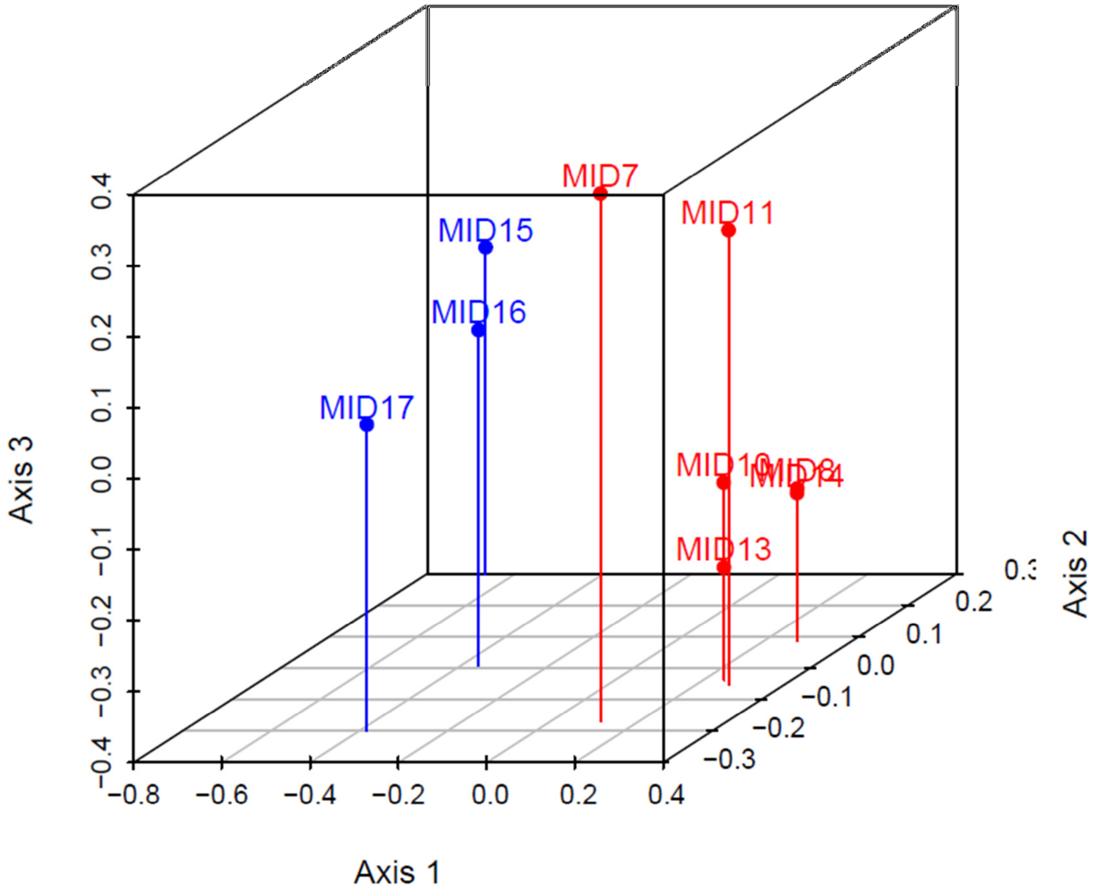

Callewaert *et al*., Figure S4. Chao1 richness estimator of the data on the complete dataset (A) and on the normalized dataset (B).

A

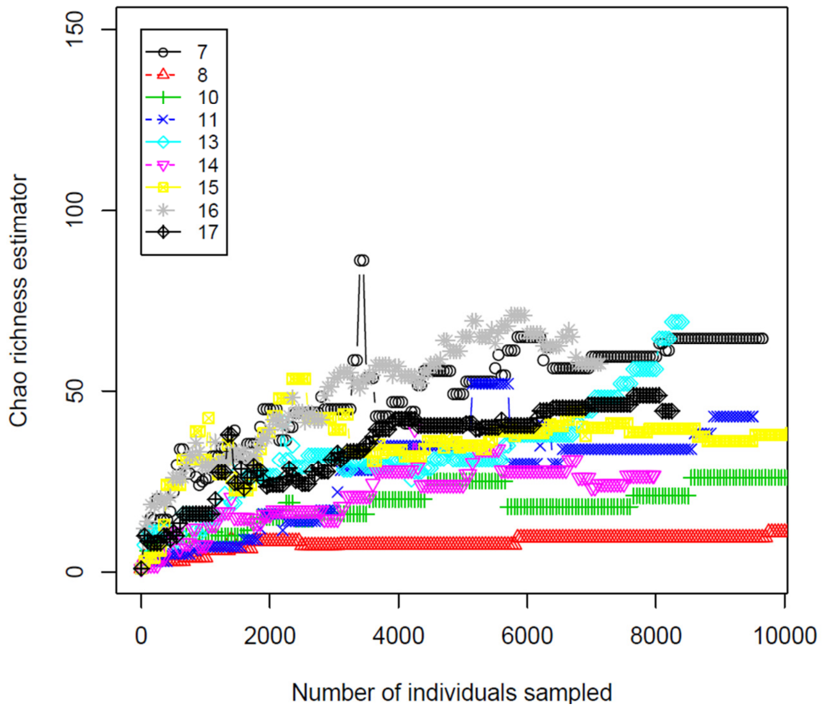

B

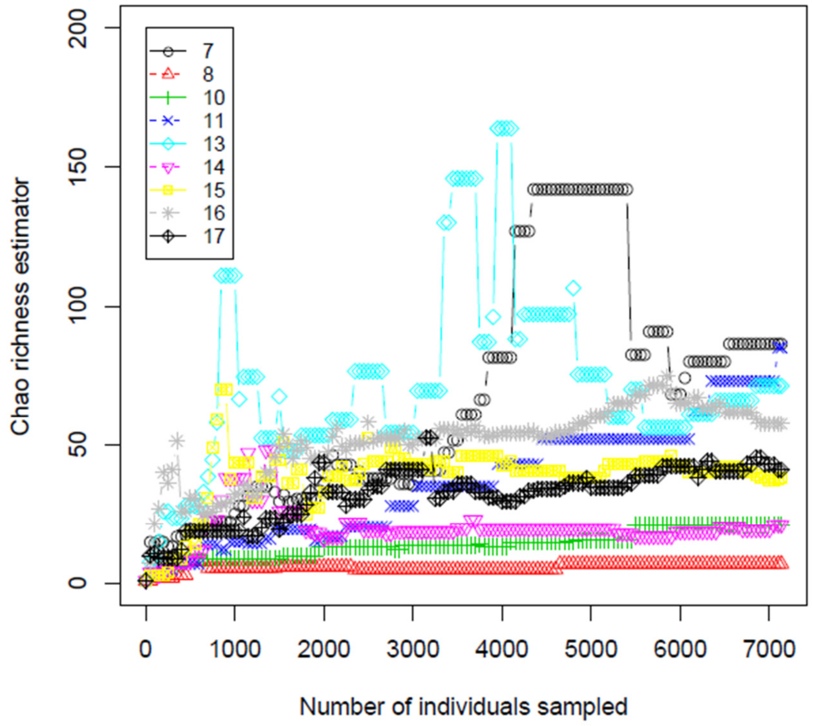

Callewaert *et al.*, Figure S5. Shannon community diversity index datacurve on the complete dataset (A) and on the normalized dataset (B). The normalized and complete dataset displayed a stable curve for the index, indicating a reliable diversity index.

A

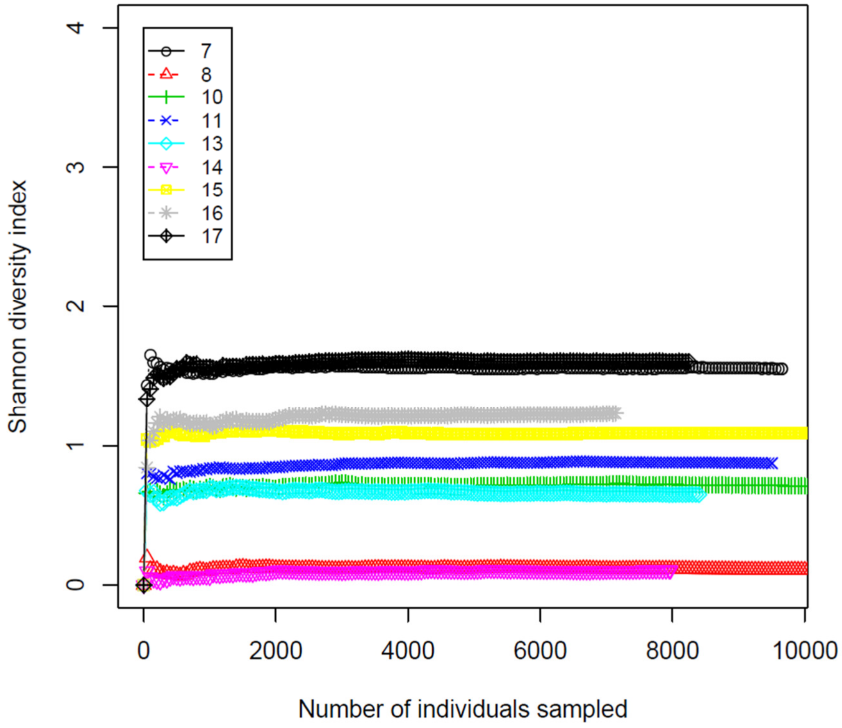

B

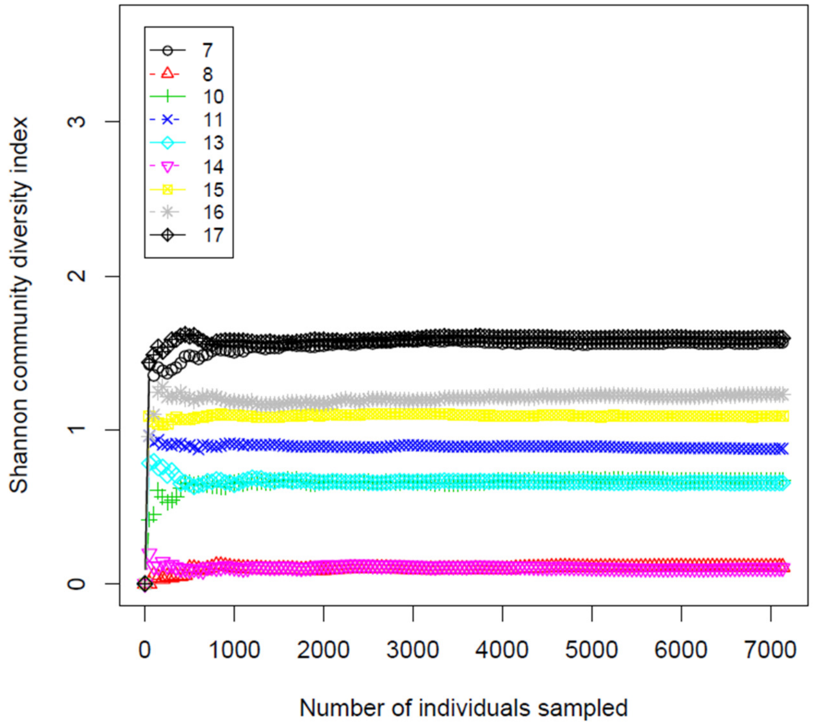

Callewaert *et al*., Figure S6. Diversity indices of the DGGE results: Range-weighted richness (Rr) – indication of species richness – and community organization (Co) – indication of species evenness – of 43 DGGE samples.

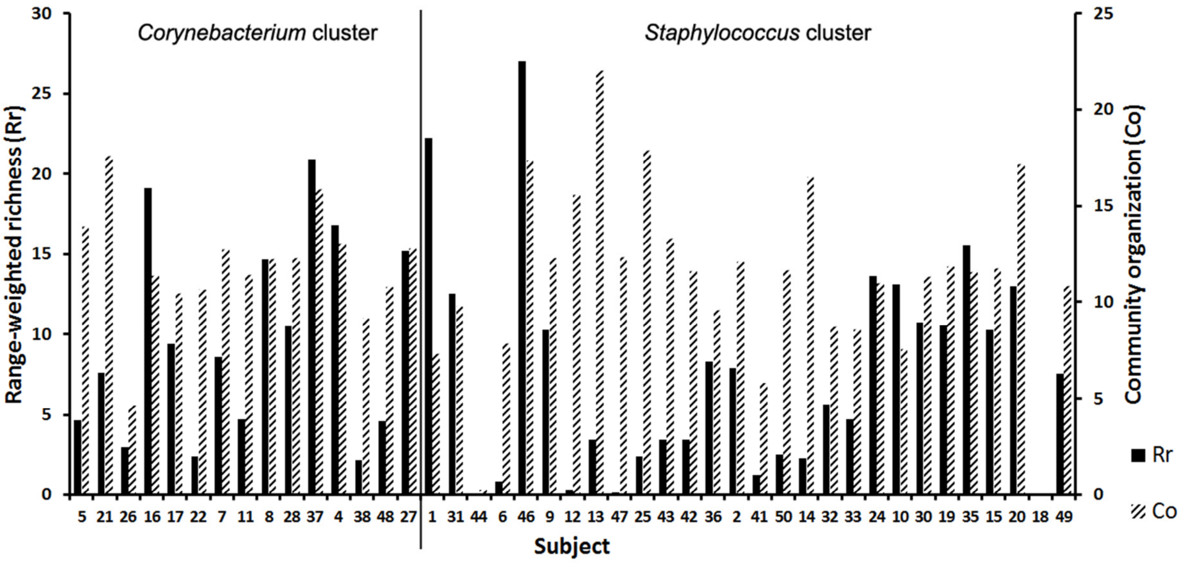

Callewaert *et al.*, Figure S7. Information and permission form for subjects giving axillary samples.

Axillary samples: Information and permission form
We will take 6 samples of your axillary bacteria by means of cotton swabs (3 left and 3 right). Your data will be handled confidentially. Research approved by the Ghent University Hospital Ethical Committee.

Person code Gender

Date & time Age

When did you last wash yourself (e.g. last night 9 pm)?

How do you wash yourself (soap, douche gel, other)?

Frequency of washing (x times/week)?

Since when do you wear your current clothes (e.g. yesterday 7 am)?

Do you use a deodorant? Y / N

If so, what is the frequency?

When did you last use it?

Brand? Roll-on or spray?

Do you have a partner? Y / N

Do you shave your armpits? Y / N

Are you left or right handed? L / R

I agree to give this information and to give bacterial samples of my axillae for further research. Y / N

Thank you for your contribution!

Callewaert *et al.*, Table S1. (A) Primers and targeted hypervariable 16S rRNA gene region for the PCR for DGGE, 454 pyrosequencing and Sanger sequencing. (B) PCR program of the PCR for DGGE, 454 pyrosequencing and Sanger sequencing.

A

**PCR**	**Primers**	**16S rRNA gene region**	**Reference**
DGGE	P338F-GC<br>P518R	V3-V4 region	[4]<br>[5]
454 pyrosequencing	530F-mod<br>1061R	V4-V6 region	[12]<br>[13]
Sanger sequencing	P63F<br>P1387R	V1-V8 region	[11]

B

**PCR**	**PCR program**
DGGE	- 10 min at 95°C<br>- 35 cycles of 1 min at 94°C, 1 min at 53°C, 2 min at 72°C<br>- 10 min at 72°C
454 pyrosequencing	- 30 sec at 98ºC<br>- 30 cycles of 5 sec at 98ºC, 20 sec at 53ºC, 20 sec at 72ºC<br>- 5 min at 72ºC
Sanger sequencing	- 10 min 95°C<br>- 35 cycles of 1 min 94°C, 1 min of 53°C, 2 min of 72°C<br>- 10 min at 72°C

Callewaert *et al.*, Table S2. Sanger sequencing identification and submission numbers of bacterial isolates on blood agar plates correlated to its denaturation place on DGGE-gel on Figure 1.

**DGGE reference**	**Identification based on BLAST**	**Max identity (%)**	**BankIt submission numbers**	**BankIt accession numbers**
A	*Staphylococcus epidermidis*	100%	1577179	KC107217
B	*Staphylococcus* spp.	99%	1577324	KC107218
C	*Staphylococcus hominis*	99%	1577325	KC107219
G	*Corynebacterium* spp.	99%	1577327	KC107220
H	*Corynebacterium* spp.	99%	1577328	KC107221

The 454 pyrosequence data are available through SRA study accession code SRP023149, or the following link: http://www.ncbi.nlm.nih.gov/sra/?term=SRP023149

Callewaert *et al.*, Table S3. Alpha diversity characteristics of the pyrosequenced samples: estimated Chao1 richness, inverse Simpson community diversity estimator, Shannon index community diversity descriptive statistics and observed richness.

Chao1 richness estimator descriptive statistics on the subsampled dataset

MID	7	8	10	11	13	14	15	16	17
Max	142	8	21	85	164	48	70	74.7	52.5
Mean	66.95	5.76	14.17	36.83	71.37	20.11	38.51	51.44	32.23
Median	61.00	5.75	13.75	35.00	66.00	19.43	40.75	53.92	34.14

Inverse Simpson community diversity estimator statistics on the subsampled dataset

	7	8	10	11	13	14	15	16	17
Max	3.19	1.04	1.39	2.41	1.51	1.09	3.05	2.38	3.86
Mean	3.11	1.03	1.37	2.15	1.36	1.03	2.53	2.30	3.59
Median	3.15	1.04	1.38	2.15	1.36	1.03	2.53	2.31	3.60

Shannon index for community diversity statistics on the subsampled dataset

	7	8	10	11	13	14	15	16	17
Max	1.58	0.12	0.68	0.96	0.80	0.20	1.11	1.28	1.62
Mean	1.54	0.10	0.65	0.89	0.66	0.11	1.08	1.20	1.58
Median	1.56	0.10	0.67	0.89	0.66	0.11	1.09	1.21	1.59

Chao1 richness estimator descriptive statistics on the subsampled dataset

	MID	nseqs	Sobs	coverage	Chao	chao_lci	chao_hci	invsimp	invs_lci	invs_hci	shann	shann_lci	shann_hci
1	MID10	7135	15	0.999439	21.00	15.94	53.25	1.38	1.35	1.41	0.67	0.64	0.70
2	MID11	7135	19	0.998318	85.00	41.10	216.07	2.13	2.11	2.16	0.88	0.86	0.90
3	MID13	7135	33	0.997477	71.25	44.59	159.21	1.36	1.33	1.38	0.66	0.63	0.69
4	MID14	7135	19	0.999159	21.14	19.34	32.39	1.03	1.02	1.03	0.10	0.08	0.12
5	MID15	7135	34	0.998879	38.00	34.81	53.80	2.53	2.48	2.58	1.09	1.07	1.11
6	MID16	7135	52	0.998458	57.50	53.35	74.43	2.33	2.28	2.38	1.23	1.20	1.26
7	MID17	7135	36	0.998598	41.00	37.16	57.57	3.58	3.49	3.67	1.59	1.57	1.62
8	MID7	7135	41	0.997617	86.33	54.09	198.03	3.18	3.11	3.26	1.57	1.54	1.60
9	MID8	7135	6	0.999720	7.00	6.07	19.66	1.04	1.03	1.05	0.11	0.09	0.12

Callewaert *et al.*, Table S4. (A) Parsimony analysis on the dendrogram data comparing the observed clusters. Significance level (α) 0.05. 10000 iterations. (B) Amova results for the Bray-Curtis distance matrix. The null-hypothesis H_0 tested is that the centroids of the clouds representing a group are statistically equal. This hypothesis is rejected by the Bray-Curtis based amova analysis. Hence, a statistically significant separation between both groups was observed.

A

Dataset	Index	p-value	Sig. difference?
Subsampled	Bray - Curtis	0.0174	Yes

B

Coryne-Staph	Among	Within	Total
SS	1.00737	0.575034	1.58241
df	1	7	8
MS	1.00737	0.0821477	
Fs:	12.2629		
p-value:	0.004*		

*significant differences among the two clusters

Callewaert *et al.*, Table S5. Used alpha diversity indices to analyze DGGE and pyrosequencing results.

**Richness**	Range-weighted richness (Rr)	DGGE specific richness estimator, correlated with the distribution of the bands in the DGGE pattern and the percentage denaturant gradient of the gel needed to represent the sample's total diversity.
	Chao1 richness estimator	A non-parametric estimator for species richness that takes the form as $S = S_{obs} + (a^2/2b)$ where S_{obs} is the number of species observed; a is the number of species observed just once; and b is the number of species observed just twice. Here used for the pyrosequencing results.
**Evenness**	Community organization (Co)	Describes the species abundance distribution in the microbial community and is calculated as the Gini coefficient times 100. Here used to describe the evenness of the DGGE samples.
**Diversity**	Shannon's diversity index	Index to describe the diversity of the community of the pyrosequencing data, which takes form as $H = \sum_{i=1}^{s} - (P_i * \ln P_i)$ with P_i = fraction of the entire population made up of species i and S = numbers of species encountered.
	Rarefaction	The rarefaction curve is plotted as the number of unique sequences detected in function of the number of total detected sequences in the sample. Used to analyze the pyrosequencing data to assess the species richness (height of the curve) and diversity (steepness of the curve).
**Dynamics**	Dynamics (Dy)	Used to determine the rate of change within the same subject over a fixed time interval of the DGGE results.

Callewaert *et al* ., Table S6. Taxonomy and size of all 159 characterized OTUs of the combined pyrosequenced dataset.

OTU	Size	Kingdom	Phylum (max identity)	Class (max identity)	Order (max identity)	Family (max identity)	Genus (max identity)
1	50445	Bacteria(100)	Actinobacteria(100)	Actinobacteria(100)	Actinomycetales(100)	Corynebacteriaceae(100)	Corynebacterium(100)
2	8999	Bacteria(100)	Proteobacteria(100)	Gammaproteobacteria(100)	Pseudomonadales(85)	Moraxellaceae(85)	unclassified(84)
3	1571	Bacteria(100)	Firmicutes(100)	Clostridia(100)	Clostridiales(100)	Incertae_Sedis_XI(100)	Peptoniphilus(100)
4	903	Bacteria(100)	Firmicutes(100)	Clostridia(100)	Clostridiales(100)	Incertae_Sedis_XI(100)	Anaerococcus(100)
5	15064	Bacteria(100)	Firmicutes(100)	Bacilli(100)	Bacillales(100)	Staphylococcaceae(100)	Staphylococcus(100)
6	849	Bacteria(100)	Firmicutes(100)	Clostridia(100)	Clostridiales(100)	Incertae_Sedis_XI(100)	Anaerococcus(100)
7	104	Bacteria(100)	Firmicutes(100)	Clostridia(100)	Clostridiales(100)	Veillonellaceae(100)	unclassified(100)
8	141	Bacteria(100)	Firmicutes(100)	Clostridia(100)	Clostridiales(100)	Incertae_Sedis_XI(100)	Finegoldia(100)
9	1596	Bacteria(100)	Actinobacteria(100)	Actinobacteria(100)	Actinomycetales(100)	Corynebacteriaceae(100)	Corynebacterium(100)
10	5	Bacteria(100)	Proteobacteria(100)	Gammaproteobacteria(100)	unclassified(100)	unclassified(100)	unclassified(100)
11	15	Bacteria(100)	Actinobacteria(100)	Actinobacteria(100)	Actinomycetales(100)	Dietziaceae(80)	Dietzia(80)
12	5	Bacteria(100)	Firmicutes(100)	Bacilli(100)	Lactobacillales(100)	Streptococcaceae(100)	Streptococcus(100)
13	6	Bacteria(100)	Proteobacteria(100)	Gammaproteobacteria(100)	unclassified(100)	unclassified(100)	unclassified(100)
14	2	Bacteria(100)	Actinobacteria(100)	Actinobacteria(100)	Actinomycetales(100)	Corynebacteriaceae(100)	Corynebacterium(100)
15	1	Bacteria(100)	Firmicutes(100)	Clostridia(100)	Clostridiales(100)	Incertae_Sedis_XI(100)	Anaerococcus(100)
16	1	Bacteria(100)	Actinobacteria(100)	Actinobacteria(100)	Actinomycetales(100)	Corynebacteriaceae(100)	Corynebacterium(100)
17	221	Bacteria(100)	Bacteroidetes(100)	Flavobacteria(100)	Flavobacteriales(100)	Flavobacteriaceae(100)	unclassified(100)
18	2	Bacteria(100)	Bacteroidetes(100)	Flavobacteria(100)	Flavobacteriales(100)	unclassified(100)	unclassified(100)
19	1	Bacteria(100)	Actinobacteria(100)	Actinobacteria(100)	Actinomycetales(100)	Corynebacteriaceae(100)	Corynebacterium(100)
20	1	Bacteria(100)	Actinobacteria(100)	Actinobacteria(100)	Actinomycetales(100)	Corynebacteriaceae(100)	Corynebacterium(100)
21	1	Bacteria(100)	Firmicutes(100)	Clostridia(100)	Clostridiales(100)	Veillonellaceae(100)	unclassified(100)
22	1	Bacteria(100)	Firmicutes(100)	Clostridia(100)	Clostridiales(100)	Veillonellaceae(100)	unclassified(100)
23	1	Bacteria(100)	Proteobacteria(100)	Gammaproteobacteria(100)	Pseudomonadales(100)	Moraxellaceae(100)	unclassified(100)
24	2	Bacteria(100)	Actinobacteria(100)	Actinobacteria(100)	Actinomycetales(100)	Corynebacteriaceae(100)	Corynebacterium(100)
25	1	Bacteria(100)	Actinobacteria(100)	Actinobacteria(100)	Actinomycetales(100)	Corynebacteriaceae(100)	Corynebacterium(100)
26	1	Bacteria(100)	Actinobacteria(100)	Actinobacteria(100)	Actinomycetales(100)	Corynebacteriaceae(100)	Corynebacterium(100)
27	1	Bacteria(100)	Actinobacteria(100)	Actinobacteria(100)	Actinomycetales(100)	Corynebacteriaceae(100)	Corynebacterium(100)
28	399	Bacteria(100)	Firmicutes(100)	Bacilli(100)	Lactobacillales(100)	Aerococcaceae(100)	Facklamia(100)
29	338	Bacteria(100)	Firmicutes(100)	Clostridia(100)	Clostridiales(100)	Incertae_Sedis_XI(100)	unclassified(100)
30	231	Bacteria(100)	Firmicutes(100)	Clostridia(100)	Clostridiales(100)	Incertae_Sedis_XI(100)	Anaerococcus(100)
31	510	Bacteria(100)	Firmicutes(100)	Clostridia(100)	Clostridiales(100)	Incertae_Sedis_XI(100)	Anaerococcus(100)
32	11	Bacteria(100)	Actinobacteria(100)	Actinobacteria(100)	Actinomycetales(100)	Dermabacteraceae(100)	Brachybacterium(100)
33	6	Bacteria(100)	Firmicutes(100)	Clostridia(100)	Clostridiales(100)	Incertae_Sedis_XI(100)	Anaerococcus(100)
34	18	Bacteria(100)	Actinobacteria(100)	Actinobacteria(100)	Actinomycetales(100)	Microbacteriaceae(100)	unclassified
35	1	Bacteria(100)	Proteobacteria(100)	Betaproteobacteria(100)	Burkholderiales(100)	unclassified(100)	unclassified(100)
36	1	Bacteria(100)	Firmicutes(100)	Clostridia(100)	Clostridiales(100)	Incertae_Sedis_XI(100)	Anaerococcus(100)
37	3	Bacteria(100)	Firmicutes(100)	Clostridia(100)	Clostridiales(100)	Incertae_Sedis_XI(100)	unclassified(100)
38	1	Bacteria(100)	Actinobacteria(100)	Actinobacteria(100)	Actinomycetales(100)	Propionibacteriaceae(100)	Brooklawnia(100)
39	1	Bacteria(100)	Actinobacteria(100)	Actinobacteria(100)	Actinomycetales(100)	Corynebacteriaceae(100)	Corynebacterium(100)
40	1	Bacteria(100)	Actinobacteria(100)	Actinobacteria(100)	Actinomycetales(100)	Corynebacteriaceae(100)	Corynebacterium(100)
41	1	Bacteria(100)	Actinobacteria(100)	Actinobacteria(100)	Actinomycetales(100)	Corynebacteriaceae(100)	Corynebacterium(100)
42	2	Bacteria(100)	Firmicutes(100)	Clostridia(100)	Clostridiales(100)	Incertae_Sedis_XI(100)	unclassified(100)
43	3	Bacteria(100)	Actinobacteria(100)	Actinobacteria(100)	Actinomycetales(100)	Corynebacteriaceae(100)	Corynebacterium(100)
44	1	Bacteria(100)	Firmicutes(100)	Clostridia(100)	Clostridiales(100)	Incertae_Sedis_XI(100)	Anaerococcus(100)
45	1	Bacteria(100)	Firmicutes(100)	Clostridia(100)	Clostridiales(100)	Incertae_Sedis_XI(100)	unclassified(100)
46	1	Bacteria(100)	Firmicutes(100)	Clostridia(100)	Clostridiales(100)	Incertae_Sedis_XI(100)	unclassified(100)
47	1	Bacteria(100)	Firmicutes(100)	Clostridia(100)	Clostridiales(100)	Incertae_Sedis_XI(100)	Anaerococcus(100)
48	1	Bacteria(100)	Actinobacteria(100)	Actinobacteria(100)	Actinomycetales(100)	unclassified(100)	unclassified(100)
49	65	Bacteria(100)	Actinobacteria(100)	Actinobacteria(100)	Actinomycetales(100)	Corynebacteriaceae(100)	Corynebacterium(100)
50	4	Bacteria(100)	Bacteroidetes(100)	Bacteroidia(100)	Bacteroidales(100)	Porphyromonadaceae(100)	Porphyromonas(100)
51	1	Bacteria(100)	Actinobacteria(100)	Actinobacteria(100)	Actinomycetales(100)	unclassified(100)	unclassified(100)
52	1	Bacteria(100)	Actinobacteria(100)	Actinobacteria(100)	Actinomycetales(100)	Corynebacteriaceae(100)	Corynebacterium(100)
53	1	Bacteria(100)	Actinobacteria(100)	Actinobacteria(100)	Actinomycetales(100)	Corynebacteriaceae(100)	Corynebacterium(100)
54	1	Bacteria(100)	Actinobacteria(100)	Actinobacteria(100)	Actinomycetales(100)	Corynebacteriaceae(100)	Corynebacterium(100)
55	4	Bacteria(100)	Proteobacteria(100)	Betaproteobacteria(100)	Burkholderiales(100)	unclassified(100)	unclassified(100)
56	20	Bacteria(100)	Proteobacteria(100)	Gammaproteobacteria(100)	Pseudomonadales(100)	Moraxellaceae(100)	Acinetobacter(100)
57	25	Bacteria(100)	Actinobacteria(100)	Actinobacteria(100)	Actinomycetales(100)	Micrococcaceae(100)	Micrococcus(100)
58	29	Bacteria(100)	Proteobacteria(100)	Alphaproteobacteria(100)	Rhodobacterales(100)	Rhodobacteraceae(100)	Paracoccus(100)
59	41	Bacteria(100)	Firmicutes(100)	Clostridia(100)	unclassified(100)	unclassified(100)	unclassified(100)
60	4	Bacteria(100)	Proteobacteria(100)	Alphaproteobacteria(100)	Rhizobiales(100)	Bradyrhizobiaceae(100)	Bosea(100)
61	4	Bacteria(100)	Proteobacteria(100)	Betaproteobacteria(100)	Burkholderiales(100)	unclassified(100)	unclassified(100)
62	1	Bacteria(100)	Actinobacteria(100)	Actinobacteria(100)	Actinomycetales(100)	Corynebacteriaceae(100)	Corynebacterium(100)
63	32	Bacteria(100)	Proteobacteria(100)	Gammaproteobacteria(100)	Pseudomonadales(100)	Moraxellaceae(100)	Enhydrobacter(100)
64	1	Bacteria(100)	Actinobacteria(100)	Actinobacteria(100)	Actinomycetales(100)	Corynebacteriaceae(100)	Corynebacterium(100)
65	1	Bacteria(100)	Proteobacteria(100)	Alphaproteobacteria(100)	Sphingomonadales(100)	Sphingomonadaceae(100)	Sphingomonas(100)
66	3	Bacteria(100)	Bacteroidetes(100)	Flavobacteria(100)	Flavobacteriales(100)	Flavobacteriaceae(100)	Chryseobacterium(100)
67	13	Bacteria(100)	Bacteroidetes(100)	Sphingobacteria(100)	Sphingobacteriales(100)	Chitinophagaceae(100)	Sediminibacterium(100)
68	2	Bacteria(100)	Actinobacteria(100)	Actinobacteria(100)	Actinomycetales(100)	Corynebacteriaceae(100)	Corynebacterium(100)
69	2857	Bacteria(100)	Proteobacteria(100)	Betaproteobacteria(100)	Burkholderiales(100)	Burkholderiales_incertae_sedis(99)	Aquabacterium(99)
70	2519	Bacteria(100)	Proteobacteria(100)	Betaproteobacteria(100)	Burkholderiales(100)	Comamonadaceae(100)	Acidovorax(92)
71	49	Bacteria(100)	Proteobacteria(100)	Gammaproteobacteria(100)	Pseudomonadales(100)	Pseudomonadaceae(100)	Pseudomonas(100)
72	10	Bacteria(100)	Deinococcus-Thermus(100)	Deinococci(100)	Deinococcales(100)	Deinococcaceae(100)	Deinococcus(100)
73	17	Bacteria(100)	Proteobacteria(100)	Gammaproteobacteria(100)	Xanthomonadales(100)	Xanthomonadaceae(100)	Stenotrophomonas(100)
74	13	Bacteria(100)	Cyanobacteria(100)	Cyanobacteria(100)	Cyanobacteria_order_incertae_sedis(100)	Chloroplast(100)	Streptophyta(100)
75	19	Bacteria(100)	Actinobacteria(100)	Actinobacteria(100)	Actinomycetales(100)	Nocardiaceae(100)	Nocardia(100)
76	7	Bacteria(100)	Actinobacteria(100)	Actinobacteria(100)	Actinomycetales(100)	Microbacteriaceae(100)	Microbacterium(100)
77	7	Bacteria(100)	Bacteroidetes(100)	Bacteroidia(100)	Bacteroidales(100)	unclassified(100)	unclassified(100)
78	5	Bacteria(100)	Actinobacteria(100)	Actinobacteria(100)	Actinomycetales(100)	Corynebacteriaceae(100)	Corynebacterium(100)
79	9	Bacteria(100)	unclassified(100)	unclassified(100)	unclassified(100)	unclassified(100)	unclassified(100)
80	5	Bacteria(100)	Proteobacteria(100)	unclassified(100)	unclassified(100)	unclassified(100)	unclassified(100)
81	5	Bacteria(100)	Proteobacteria(100)	Betaproteobacteria(100)	Rhodocyclales(100)	Rhodocyclaceae(100)	unclassified(100)
82	3	Bacteria(100)	Proteobacteria(100)	Betaproteobacteria(100)	Burkholderiales(100)	Burkholderiaceae(100)	Ralstonia(100)
83	5	Bacteria(100)	Proteobacteria(100)	Gammaproteobacteria(100)	Pasteurellales(100)	Pasteurellaceae(100)	Actinobacillus(100)
84	17	Bacteria(100)	Proteobacteria(100)	Gammaproteobacteria(100)	Enterobacteriales(100)	Enterobacteriaceae(100)	Escherichia/Shigella(100)
85	4	Bacteria(100)	Firmicutes(100)	Clostridia(100)	Clostridiales(100)	Lachnospiraceae(100)	Catonella(100)
86	2	Bacteria(100)	Proteobacteria(100)	Gammaproteobacteria(100)	Enterobacteriales(100)	Enterobacteriaceae(100)	Citrobacter(100)
87	3	Bacteria(100)	Cyanobacteria(100)	Cyanobacteria(100)	Cyanobacteria_order_incertae_sedis(100)	Family_I(100)	GpI(100)
88	24	Bacteria(100)	Proteobacteria(100)	Alphaproteobacteria(100)	Rhodobacterales(100)	Rhodobacteraceae(100)	Paracoccus(100)
89	9	Bacteria(100)	Actinobacteria(100)	Actinobacteria(100)	Actinomycetales(100)	Nocardiaceae(100)	Nocardia(100)

90	1	Bacteria(100)	Proteobacteria(100)	Betaproteobacteria(100)	Burkholderiales(100)	Comamonadaceae(100)	Comamonas(100)
91	1	Bacteria(100)	Proteobacteria(100)	Betaproteobacteria(100)	Burkholderiales(100)	Comamonadaceae(100)	unclassified(100)
92	6	Bacteria(100)	Actinobacteria(100)	Actinobacteria(100)	Actinomycetales(100)	Sanguibacteraceae(84)	Sanguibacter(84)
93	1	Bacteria(100)	Proteobacteria(100)	Betaproteobacteria(100)	Burkholderiales(100)	Comamonadaceae(100)	unclassified(100)
94	1	Bacteria(100)	Firmicutes(100)	Bacilli(100)	Bacillales(100)	Bacillaceae(100)	Geobacillus(100)
95	1	Bacteria(100)	Bacteroidetes(100)	Sphingobacteria(100)	Sphingobacteriales(100)	Chitinophagaceae(100)	Chitinophaga(100)
96	23	Bacteria(100)	Firmicutes(100)	Bacilli(100)	Lactobacillales(100)	Lactobacillaceae(100)	Lactobacillus(100)
97	6	Bacteria(100)	Firmicutes(100)	Erysipelotrichi(100)	Erysipelotrichales(100)	Erysipelotrichaceae(100)	Bulleidia(100)
98	6	Bacteria(100)	Actinobacteria(100)	Actinobacteria(100)	Actinomycetales(100)	Nocardioidaceae(100)	Aeromicrobium(100)
99	7	Bacteria(100)	Bacteroidetes(100)	Sphingobacteria(100)	Sphingobacteriales(100)	Chitinophagaceae(100)	Ferruginibacter(100)
100	8	Bacteria(100)	Proteobacteria(100)	Betaproteobacteria(100)	Burkholderiales(100)	Oxalobacteraceae(100)	unclassified(100)
101	11	Bacteria(100)	Proteobacteria(100)	Betaproteobacteria(100)	Rhodocyclales(100)	Rhodocyclaceae(100)	unclassified(100)
102	6	Bacteria(100)	Proteobacteria(100)	Gammaproteobacteria(100)	Enterobacteriales(100)	Enterobacteriaceae(100)	Serratia(100)
103	3	Bacteria(100)	Planctomycetes(100)	Planctomycetacia(100)	Planctomycetales(100)	Planctomycetaceae(100)	Singulisphaera(100)
104	5	Bacteria(100)	Bacteroidetes(100)	Bacteroidia(100)	Bacteroidales(100)	Bacteroidaceae(100)	Bacteroides(100)
105	2	Bacteria(100)	Firmicutes(100)	unclassified(100)	unclassified(100)	unclassified(100)	unclassified(100)
106	2	Bacteria(100)	Firmicutes(100)	Clostridia(100)	Clostridiales(100)	Clostridiaceae(100)	Clostridium(100)
107	2	Bacteria(100)	unclassified(100)	unclassified(100)	unclassified(100)	unclassified(100)	unclassified(100)
108	2	Bacteria(100)	Proteobacteria(100)	Alphaproteobacteria(100)	Caulobacterales(100)	Caulobacteraceae(100)	Brevundimonas(100)
109	2	Bacteria(100)	Bacteroidetes(100)	Bacteroidia(100)	Bacteroidales(100)	Prevotellaceae(100)	Prevotella(100)
110	2	Bacteria(100)	Proteobacteria(100)	Alphaproteobacteria(100)	Caulobacterales(100)	Caulobacteraceae(100)	Phenylobacterium(100)
111	3	Bacteria(100)	Actinobacteria(100)	Actinobacteria(100)	Actinomycetales(100)	Micrococcaceae(100)	Kocuria(100)
112	1	Bacteria(100)	Firmicutes(100)	Clostridia(100)	Clostridiales(100)	Incertae_Sedis_XI(100)	Anaerococcus(100)
113	1	Bacteria(100)	Actinobacteria(100)	Actinobacteria(100)	Actinomycetales(100)	Propionibacteriaceae(100)	Propionibacterium(100)
114	1	Bacteria(100)	Actinobacteria(100)	Actinobacteria(100)	Actinomycetales(100)	unclassified(100)	unclassified(100)
115	2	Bacteria(100)	Proteobacteria(100)	Betaproteobacteria(100)	Rhodocyclales(100)	Rhodocyclaceae(100)	unclassified
116	1	Bacteria(100)	Firmicutes(100)	Clostridia(100)	unclassified(100)	unclassified(100)	unclassified(100)
117	1	Bacteria(100)	Proteobacteria(100)	unclassified(100)	unclassified(100)	unclassified(100)	unclassified(100)
118	1	Bacteria(100)	Proteobacteria(100)	Alphaproteobacteria(100)	Sphingomonadales(100)	Sphingomonadaceae(100)	Sphingobium(100)
119	1	Bacteria(100)	Proteobacteria(100)	Alphaproteobacteria(100)	Rhodospirillales(100)	Acetobacteraceae(100)	Roseomonas(100)
120	1	Bacteria(100)	Proteobacteria(100)	Alphaproteobacteria(100)	Sphingomonadales(100)	Sphingomonadaceae(100)	Sphingomonas(100)
121	1	Bacteria(100)	Actinobacteria(100)	Actinobacteria(100)	Actinomycetales(100)	Corynebacteriaceae(100)	Corynebacterium(100)
122	1	Bacteria(100)	Proteobacteria(100)	Alphaproteobacteria(100)	Rhodospirillales(100)	Acetobacteraceae(100)	unclassified(100)
123	3	Bacteria(100)	Proteobacteria(100)	Alphaproteobacteria(100)	Rhizobiales(100)	Phyllobacteriaceae(100)	Aminobacter(100)
124	2	Bacteria(100)	Firmicutes(100)	Clostridia(100)	Clostridiales(100)	Incertae_Sedis_XI(100)	Anaerococcus(100)
125	2	Bacteria(100)	Firmicutes(100)	Bacilli(100)	Lactobacillales(100)	Streptococcaceae(100)	Streptococcus(100)
126	1	Bacteria(100)	Proteobacteria(100)	Betaproteobacteria(100)	Burkholderiales(100)	Oxalobacteraceae(100)	Collimonas(100)
127	2	Bacteria(100)	Firmicutes(100)	Clostridia(100)	Clostridiales(100)	Incertae_Sedis_XI(100)	Anaerococcus(100)
128	1	Bacteria(100)	Firmicutes(100)	Bacilli(100)	Lactobacillales(100)	unclassified(100)	unclassified(100)
129	2	Bacteria(100)	Actinobacteria(100)	Actinobacteria(100)	Actinomycetales(100)	Nocardiaceae(100)	Rhodococcus(100)
130	1	Bacteria(100)	Proteobacteria(100)	Alphaproteobacteria(100)	Caulobacterales(100)	Caulobacteraceae(100)	Phenylobacterium(100)
131	2	Bacteria(100)	Actinobacteria(100)	Actinobacteria(100)	Actinomycetales(100)	Streptomycetaceae(100)	Streptomyces(100)
132	1	Bacteria(100)	Firmicutes(100)	Clostridia(100)	Clostridiales(100)	Incertae_Sedis_XI(100)	Peptoniphilus(100)
133	1	Bacteria(100)	Proteobacteria(100)	Alphaproteobacteria(100)	Rhizobiales(100)	Bradyrhizobiaceae(100)	unclassified(100)
134	1	Bacteria(100)	Actinobacteria(100)	Actinobacteria(100)	Actinomycetales(100)	Corynebacteriaceae(100)	Corynebacterium(100)
135	1	Bacteria(100)	Actinobacteria(100)	Actinobacteria(100)	Actinomycetales(100)	Corynebacteriaceae(100)	Corynebacterium(100)
136	1	Bacteria(100)	Proteobacteria(100)	Betaproteobacteria(100)	Neisseriales(100)	Neisseriaceae(100)	Neisseria(100)
137	105	Bacteria(100)	Firmicutes(100)	Clostridia(100)	Clostridiales(100)	Incertae_Sedis_XI(98)	unclassified(98)
138	12	Bacteria(100)	Actinobacteria(100)	Actinobacteria(100)	Actinomycetales(100)	Corynebacteriaceae(100)	Corynebacterium(100)
139	9	Bacteria(100)	Proteobacteria(100)	Epsilonproteobacteria(100)	Campylobacterales(100)	Campylobacteraceae(100)	Campylobacter(100)
140	2	Bacteria(100)	Acidobacteria(100)	Acidobacteria_Gp3(100)	unclassified(100)	unclassified(100)	unclassified(100)
141	2	Bacteria(100)	Proteobacteria(100)	unclassified(100)	unclassified(100)	unclassified(100)	unclassified(100)
142	2	Bacteria(100)	Firmicutes(100)	Bacilli(100)	Lactobacillales(100)	Aerococcaceae(100)	Aerococcus(100)
143	1	Bacteria(100)	Actinobacteria(100)	Actinobacteria(100)	Actinomycetales(100)	Brevibacteriaceae(100)	Brevibacterium(100)
144	1	Bacteria(100)	Actinobacteria(100)	Actinobacteria(100)	Actinomycetales(100)	unclassified(100)	unclassified(100)
145	1	Bacteria(100)	Proteobacteria(100)	Gammaproteobacteria(100)	unclassified(100)	unclassified(100)	unclassified(100)
146	1	Bacteria(100)	Proteobacteria(100)	Alphaproteobacteria(100)	Caulobacterales(100)	Caulobacteraceae(100)	Phenylobacterium(100)
147	2	Bacteria(100)	Actinobacteria(100)	Actinobacteria(100)	Actinomycetales(100)	Corynebacteriaceae(100)	Corynebacterium(100)
148	1	Bacteria(100)	Actinobacteria(100)	Actinobacteria(100)	Actinomycetales(100)	Intrasporangiaceae(100)	Janibacter(100)
149	1	Bacteria(100)	Proteobacteria(100)	Alphaproteobacteria(100)	Rhizobiales(100)	Bradyrhizobiaceae(100)	Bradyrhizobium(100)
150	1	Bacteria(100)	Proteobacteria(100)	Gammaproteobacteria(100)	unclassified(100)	unclassified(100)	unclassified(100)
151	2	Bacteria(100)	Actinobacteria(100)	Actinobacteria(100)	Actinomycetales(100)	Dermabacteraceae(100)	Dermabacter(100)
152	1	Bacteria(100)	Proteobacteria(100)	Gammaproteobacteria(100)	Pseudomonadales(100)	Moraxellaceae(100)	unclassified(100)
153	1	Bacteria(100)	Actinobacteria(100)	Actinobacteria(100)	Actinomycetales(100)	Brevibacteriaceae(100)	Brevibacterium(100)
154	1	Bacteria(100)	Proteobacteria(100)	Gammaproteobacteria(100)	unclassified(100)	unclassified(100)	unclassified(100)
155	1	Bacteria(100)	Firmicutes(100)	Bacilli(100)	Bacillales(100)	Staphylococcaceae(100)	unclassified(100)
156	48	Bacteria(100)	Spirochaetes(100)	Spirochaetes(100)	Spirochaetales(100)	Leptospiraceae(100)	Leptospira(100)
157	1	Bacteria(100)	Firmicutes(100)	Clostridia(100)	Clostridiales(100)	Incertae_Sedis_XI(100)	Anaerococcus(100)
158	1	Bacteria(100)	Actinobacteria(100)	Actinobacteria(100)	Actinomycetales(100)	Corynebacteriaceae(100)	Corynebacterium(100)
159	1	Bacteria(100)	Actinobacteria(100)	Actinobacteria(100)	Actinomycetales(100)	Corynebacteriaceae(100)	Corynebacterium(100)

Callewaert *et al* ., Table S7. Axillary samples of the subjects throughout time, with metadata concerning deodorant usage, washing behavior, handedness, armpit shaving, and other personal data.

Subject	Gender	Age	Handed-ness	Wash /week	Deo /week	Armpit shaving?	Wash	Deodorant	Cluster	Sample date	Time between last wash and sampling	Time between deodorant and sampling	Time between fresh clothes and sampling	Sample date	Time between last wash and sampling	Time between deodorant and sampling	Time between fresh clothes and sampling
1	M	35	R	6.5	7	N	Soap	spray	Staph	19/04/2011	9	9	33	25/05/2011	33	33	49
2	F	26	R	7	7	Y	Showergel	Sanex spray	Staph	19/04/2011	11.2	11.2	11.2	24/05/2011	9.4	9.4	33.4
3	M	29	R	4	5	N	Showergel	spray	Staph	19/04/2011	23.4	71.4	35	10/05/2011	8.5	8.5	32.5
4	M	24	R	3	0	N	Soap	/	Coryne	2/02/2011	42	/	42	9/02/2011	15	/	37
4										19/04/2011	23.5	/	10	20/04/2011	55	/	55
5	M	24	R	3	4	N	Showergel	Axe spray	Coryne	20/04/2011	4	4	4	19/01/2012	72.75	120.75	48.75
6	M	23	R	7	7	N	Soap	Axe spray	Staph	19/04/2011	6.5	6.5	6.5	24/05/2011	8.5	8.5	8.5
7	M	28	R	7	2	N	Soap	Roll-on	Coryne	19/04/2011	6.7	6.7	30.7				
8	M	30	R	3.5	0	N	Soap	/	Coryne	19/04/2011	55.75	/	52.75	24/05/2011	47.65	/	33.65
9	M	33	L	4	3	N	Showergel	Roll-on	Staph	19/04/2011	21	31	7.5	2/04/2012	24	129.5	48
10	F	51	R	7	5	Y	Soap	spray	Staph	19/04/2011	7.5	7.5	7.5	10/05/2011	10	34	10
11	M	27	R	8	5	N	Showergel	spray	Coryne	19/04/2011	4.3	52.3	28.3	10/05/2011	9.8	177.8	9.8
12	M	32	R	7	7	N	Soap	Axe excite	Staph	19/04/2011	4	4	4	7/06/2011	21.5	10.5	10.5
13	M	28	R	7	10	N	Soap	spray	Staph	19/04/2011	15	4	4h	7/06/2011	19	7	19
14	M	28	R	8	8	N	Showergel	Nivea spray	Staph	19/04/2011	7.5	7.5	7.5	6/01/2012	7.85	7.85	7.85
15	M	24	R	3.5	7	N	Showergel	Nivea spray	Staph	19/04/2011	38	7	37	24/05/2011	10.75	10.75	34.75
16	F	34	R	6	24	Y	Showergel	Dove spray	Coryne	19/04/2011	8.2	4.7	8.2	7/06/2011	18.5	0.5	7
17	M	28	L	7	7	N	Showergel	spray	Coryne	26/04/2011	5.5	5.25	5.5	17/08/2011	7.5	7.5	7.5
18	M	27	R	3	7	N	Showergel	Rexona spray	Staph	19/04/2011	17.4	6.4	30.4				
19	M	28	R	14	7	Y	Showergel	Sanex roll-on	Staph	19/04/2011	8	8	8	25/05/2011	9	9	9
20	F	26	R	10.5	7	Y	Soap	Dove spray	Staph	26/04/2011	5.5	5.25	24.5	17/08/2011	8.25	8	32.5
21	M	29	R	3.5	4.5	N	Showergel	Axe / Nivea spray	Coryne	23/07/2011	7.5	7.5	55.5	18/08/2011	8.75	8.75	56.75
21										24/09/2011	8.75	8.75	8.75	22/10/2011	7.5	7.25	31.5
22	M	29	R	7	7	N	Showergel	Axe spray	Coryne	10/05/2011	8.5	8.3	6.5	18/08/2011	20.5	9.5	9.5
23	M	26	R	7	14	N	Showergel	Axe spray	Staph	10/05/2011	7.75	2.75	7.75				
24	F	26	L	7	7	Y	Showergel	Nivea spray	Staph	10/05/2011	9	9	33	18/08/2011	8.75	8.75	8.75
25	M	23	L	3.5	7	N	Showergel	Axe spray	Staph	10/05/2011	8	8	46	17/08/2011	20.5	9.5	9.5
26	M	24	R	9	7	N	Soap	Adidas Ice Dive	Coryne	10/05/2011	8	8	54	18/08/2011	7.75	7.75	7.75
27	M	27	R	3	3	N	Showergel	Axe spray	Coryne	21/05/2011	21	93	93				
28	M	24	R	3	7	N	Showergel	Dove spray	Coryne	21/05/2011	20.5	20.5	20.5				
29	M	32	R	7	7	N	Showergel	Roll-on	n.d.	24/05/2011	8	7.8	7.7				
30	M	30	R	8.5	10	N	Showergel	Nivea roll-on	Staph	26/04/2011	6	6	6	25/05/2011	9.5	9.5	9.5
31	M	29	R	7	10	Y	Showergel	Axe / Adidas spray	Staph	7/06/2011	21.5	21.5	21.5	17/08/2011	17	8	32
32	F	36	R	7	1.5	Y	Showergel	Louis Widmer spray	Staph	7/06/2011	10.75	34.75	10.75				
33	M	24	R	7	7	N	Showergel	spray	Staph	10/05/2011	8	8	7.75	6/01/2012	7.5	32	7.5
34	M	32	R	6.5	4.5	N	Soap	Armani spray	Staph	10/05/2011	8	8	7.75				
35	F	44	R	7	3	Y	Soap	Roll-on	Staph	24/05/2011	8.85	32.85	8.85	17/08/2011	9	9	33
36	F	24	R	7	10	Y	Showergel	Nivea roll-on	Staph	19/04/2011	4	4	4	24/05/2011	9.5	9.25	9.25
37	F	28	R	7	5	Y	Showergel	Dove roll-on	Coryne	20/04/2011	2.5	2.5	2.5				
38	M	25	R	5	3	N	Showergel	Axe spray	Coryne	2/02/2011	34	34	34				
39	M	29	R	6	7	N	Soap	Roll-on	n.d.	17/08/2011	19.5	7.5	7.5				
40	M	25	L	5	7	Y	Showergel	Nivea roll-on	n.d.	17/08/2011	21.5	8.5	8.5				
41	F	26	R	4	7	Y	Showergel	Body shop roll-on	Staph	17/08/2011	43.5	8.5	32.5				
42	F	26	R	7	7	Y	Showergel	Roll-on	Staph	17/08/2011	9	9	9				
43	F	21	R	10	7	Y	Showergel	spray	Staph	17/08/2011	9	1	33				
44	F	36	R	7	14	Y	Showergel	Eau d'Issey	Staph	17/08/2011	8.2	8.2	8.2	2/04/2012	9.5	9.5	9.5
45	F	27	R	4.5	7	Y	Showergel	Nivea roll-on	n.d.	17/08/2011	56	10	10				
46	F	32	L	7	5	Y	Showergel	spray	Staph	17/08/2011	8	32	8	2/04/2012	9	177	8.5
47	M	29	L	7	7	Y	Showergel	Axe spray	Staph	17/08/2011	8	8	8				
48	M	65	R	2	0	N	Soap	/	Coryne	17/08/2011	94	/	58				
49	M	22	R	4	10	N	Showergel	Axe spray	Staph	17/08/2011	9	8	21	6/01/2012	19.85	7.85	18.85
50	M	27	R	6.5	5	N	Soap	Fa aqua spray	Staph	15/09/2011	15.5	7	56	12/01/2012	34.15	10.25	34.15
51	F	30	R	7	3.5	Y	Shampoo	spray	n.d.	17/08/2011	7.75	5.75	5.75				
52	M	26	R	5	0	N	Showergel	/	Staph	21/09/2011	7.5	/	31				
53	F	27	R	14	14	Y	Soap	Roll-on	Staph	22/10/2011	21	189	20.5	29/10/2011	9.5	81.5	32

*n.d. = not determined

Callewaert *et al* ., Table S7. Axillary samples of the subjects throughout time, with metadata concerning deodorant usage, washing behavior, handedness, armpit shaving, and other personal data.

Code	Sample date	Time between last wash and sampling	Time between deodorant and sampling	Time between fresh clothes and sampling	Sample date	Time between last wash and sampling	Time between deodorant and sampling	Time between fresh clothes and sampling	Sample date	Time between last wash and sampling	Time between deodorant and sampling	Time between fresh clothes and sampling
1	17/08/2011	10	10	82	6/01/2012	31	31	7				
2												
3												
4	3/03/2011	56	/	56	30/03/2011	76	/	76	7/04/2011	23.75	/	47.75
4	7/06/2011	42	/	42	18/08/2011	116	/	11	14/02/2012	56	/	32
5												
6	17/08/2011	6.5	6.5	6.5	6/01/2012	6.75	6.75	31.75				
7												
8	2/04/2012	27.4	/	10.4								
9												
10	24/05/2011	8.7	8.7	33.2	6/01/2012	9	33	9	2/04/2012	20.5	164.5	9.5
11	24/05/2011	9	9	34	17/08/2011	7.5	7.5	31.5	6/01/2012	6.5	78.5	30.5
12												
13	17/08/2011	19.75	7.75	7.75								
14	16/02/2012	9.75	81.75	9.75								
15												
16												
17												
18												
19												
20												
21	26/08/2011	33	81	81	8/09/2011	9	9	33	18/09/2011	8	8	8
21	28/10/2011	7.75	7.75	7.75	6/11/2011	8	7.75	56	21/01/2012	7.5	7.25	31.25
22	6/01/2012	6.5	6.5	30.5	12/01/2012	18	10	34	16/02/2012	10	9.5	34
23												
24	6/01/2012	8	7	31								
25	6/01/2012	42	6	30	16/02/2012	8	8	32				
26	12/01/2012	9	9	9								
27												
28												
29												
30												
31	12/01/2012	20	4	34								
32												
33	2/04/2012	10	10	10								
34												
35												
36	17/08/2011	7.5	7.5	7	2/04/2012	8.5	32.5	8.25				
37												
38												
39												
40												
41												
42												
43												
44												
45												
46												
47												
48												
49	2/04/2012	4	9.5	57.5								
50												
51												
52												
53	4/11/2011	25	73	97	12/11/2011	4	52	25	18/11/2011	14	28	13

Callewaert *et al* ., Table S8. Axillary samples of the subjects throughout time, with quantitatively and qualitatively determined left right similarity.

Subject	Gender	Age	Deo/week	Cluster	Sample date	Same bands present?	Left right similarity (%)	Sample date	Same bands present?	Left right similarity (%)	Sample date	Same bands present?	Left right similarity (%)	Sample date	Same bands present?	Left right similarity (%)	Sample date	Same bands present?	Left right similarity (%)	Average LR similarity (%)
1	M	35	7	Staph	19/04/2011	N	81.46	25/05/2011	N	56.07	17/08/2011	N	79.67	6/01/2012	N	73.99				72.80
2	F	26	7	Staph	19/04/2011	N	77.05	24/05/2011	Y	96.98										87.02
3	M	29	5	Staph	19/04/2011	Y	98.71	10/05/2011		n.d.										98.71
4	M	24	0	Coryne	2/02/2011	N	75.13	9/02/2011	N	86.04	3/03/2011	N	97.49	30/03/2011	N	95.38	7/04/2011	N	n.d.	
					19/04/2011	N	88.26	20/04/2011	N	71.76	7/06/2011	N	91.09	18/08/2011	Y	96.45	14/02/2012	Y	99.17	89.35
5	M	24	4	Coryne	20/04/2011	Y	97.87	19/01/2012	Y	81.10										89.49
6	M	23	7	Staph	19/04/2011	Y	95.92	24/05/2011	Y	97.51	17/08/2011	Y	n.d.	6/01/2012		n.d.				96.72
7	M	28	2	Coryne	19/04/2011	Y	94.93													94.93
8	M	30	0	Coryne	19/04/2011	N	96.99	24/05/2011	N	96.29										96.64
9	M	33	3	Staph	19/04/2011	Y	94.57													94.57
10	F	51	5	Staph	19/04/2011	Y	96.13	10/05/2011	Y	94.76	24/05/2011	Y	96.76	6/01/2012	N	87.31				93.74
11	M	27	5	Coryne	19/04/2011	Y	94.23	10/05/2011	N	90.30	24/05/2011	N	84.46	17/08/2011	Y	93.96	6/01/2012		n.d.	90.74
12	M	32	7	Staph	19/04/2011	Y	97.35	7/06/2011	Y	98.86										98.11
13	M	28	10	Staph	19/04/2011	Y	94.37	7/06/2011	Y	96.41	17/08/2011	Y	94.25							95.01
14	M	28	8	Staph	19/04/2011	N	85.67	6/01/2012		n.d.	16/02/2012	N	58.06							71.87
15	M	24	7	Staph	19/04/2011	N	70.09	24/05/2011	N	87.93										79.01
16	F	34	24	Coryne	19/04/2011	Y	98.49	7/06/2011	Y	90.62										94.56
17	M	28	7	Coryne	26/04/2011	N	58.91	17/08/2011	Y	90.62										74.77
18	M	27	7	Staph	19/04/2011	Y	97.32													97.32
19	M	28	7	Staph	19/04/2011	Y	83.95	25/05/2011	Y	48.15										66.05
20	F	26	7	Staph	26/04/2011	N	92.00	17/08/2011	Y	95.38										93.69
21	M	29	4.5	Coryne	10/05/2011	N	85.31	18/08/2011	N	84.61	16/02/2012	Y	97.13	3/03/2011		n.d.	30/03/2011		n.d.	89.02
22	M	29	7	Coryne	10/05/2011	N	76.93	18/08/2011	N	79.43	6/01/2012			12/01/2012		n.d.	16/02/2012		n.d.	78.18
23	M	26	14	Staph	10/05/2011	N	76.11													76.11
24	F	26	7	Staph	10/05/2011	N	84.12	18/08/2011	Y	86.51	6/01/2012	Y	91.48							87.37
25	M	23	7	Staph	10/05/2011	Y	99.39	17/08/2011		n.d.	6/01/2012		n.d.	12/01/2012		n.d.	16/02/2012	N	94.31	96.85
26	M	24	7	Coryne	10/05/2011	N	81.09	18/08/2011	N	32.75	12/01/2012	N	37.81							50.55
27	M	27	3	Coryne	21/05/2011	N	72.41													72.41
28	M	24	7	Coryne	21/05/2011	N	65.86													65.86
29	M	32	7	n.d.	24/05/2011	N	68.13													68.13
30	M	30	10	Staph	26/04/2011	Y	97.87	25/05/2011	N	88.35										93.11
31	M	29	10	Staph	7/06/2011	Y	98.29	17/08/2011	Y	81.45	12/01/2012	Y	97.88							92.54
32	F	36	1.5	Staph	7/06/2011	N	79.87													79.87
33	M	24	7	Staph	10/05/2011	Y	98.58	6/01/2012		n.d.										98.58
34	M	32	4.5	Staph	10/05/2011	Y	95.59													95.59
35	F	44	3	Staph	24/05/2011	N	81.98	17/08/2011		n.d.										81.98
36	F	24	10	Staph	19/04/2011	Y	92.87	24/05/2011	Y	n.d.	17/08/2011		n.d.							92.87
37	F	28	5	Coryne	20/04/2011	N	89.23													89.23
38	M	25	3	Coryne	2/02/2011	Y	98.54													98.54
39	M	29	7	n.d.	17/08/2011		n.d.													n.d.
40	M	25	7	n.d.	17/08/2011		n.d.													n.d.
41	F	26	7	Staph	17/08/2011	N	74.41													74.41
42	F	26	7	Staph	17/08/2011		n.d.													n.d.
43	F	21	7	Staph	17/08/2011	N	71.06													71.06
44	F	36	14	Staph	17/08/2011	Y	97.09													97.09
45	F	27	7	n.d.	17/08/2011		n.d.													n.d.
46	F	32	5	Staph	17/08/2011	Y	86.35													86.35
47	M	29	7	Staph	17/08/2011	Y	98.20													98.20
48	M	65	0	Coryne	17/08/2011	Y	94.13													94.13
49	M	22	10	Staph	17/08/2011	Y	94.89	6/01/2012	N	80.16										87.53
50	M	27	5	Staph	15/09/2011	Y	91.39	12/01/2012	Y	96.09										93.74
51	F	30	3.5	n.d.	17/08/2011		n.d.													n.d.
52	M	26	0	Staph	21/09/2011	Y	95.89													95.89
53	F	27	14	Staph																n.d.

Average	86.81	±	11.28
Staph	88.51	±	10.65
Coryne	84.22	±	8.78

*n.d. = not determined
